# Selective Enrichment of Histidine Phosphorylated Peptides Using Molecularly Imprinted Polymers

**DOI:** 10.1021/acs.analchem.0c04474

**Published:** 2021-02-16

**Authors:** Anıl Incel, Ignacio Arribas Díez, Celina Wierzbicka, Katarzyna Gajoch, Ole N. Jensen, Börje Sellergren

**Affiliations:** † Department of Biomedical Science, Faculty of Health and Society, Malmö University, 205 06 Malmö, Sweden; ‡ Department of Biochemistry & Molecular Biology and VILLUM Center for Bioanalytical Sciences, University of Southern Denmark, DK-5230 Odense M, Denmark

## Abstract

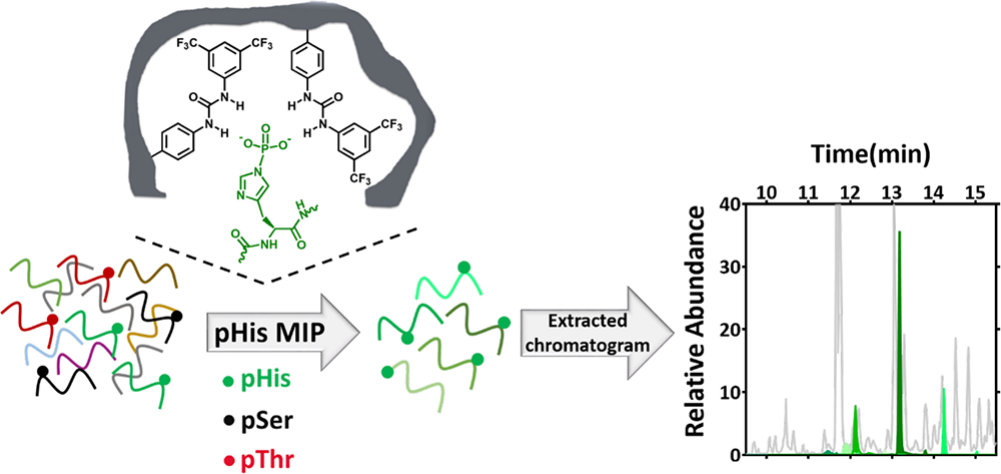

Protein histidine phosphorylation (pHis) is involved in molecular signaling networks in bacteria, fungi, plants, and higher eukaryotes including mammals and is implicated in human diseases such as cancer. Detailed investigations of the pHis modification are hampered due to its acid-labile nature and consequent lack of tools to study this post-translational modification (PTM). We here demonstrate three molecularly imprinted polymer (MIP)-based reagents, MIP1–MIP3, for enrichment of pHis peptides and subsequent characterization by chromatography and mass spectrometry (LC–MS). The combination of MIP1 and β-elimination provided some selectivity for improved detection of pHis peptides. MIP2 was amenable to larger pHis peptides, although with poor selectivity. Microsphere-based MIP3 exhibited improved selectivity and was amenable to enrichment and detection by LC–MS of pHis peptides in tryptic digests of protein mixtures. These MIP protocols do not involve any acidic solvents during sample preparation and enrichment, thus preserving the pHis modification. The presented proof-of-concept results will lead to new protocols for highly selective enrichment of labile protein phosphorylations using molecularly imprinted materials.

## Introduction

Protein phosphorylation is one of the most widely studied and best-understood post-translational modifications (PTMs). It is involved in the regulation of vital biological processes, including cellular signal transduction.^1^ Although phosphorylation can occur on at least nine different amino acid residues, i.e., serine, threonine, tyrosine, histidine, lysine, arginine, aspartate, glutamate, and cysteine, the vast majority of phosphoproteomics research is focused on the phosphorylation of the former three residues. In recent years, however, the role of histidine phosphorylation (pHis) has gained attention. This modification is well known to be involved in two-component protein-signaling networks in prokaryotes and lower eukaryotes^2−4^ and is also found in mammals and implicated in certain human disease states.^5−7^

**Figure 1:**
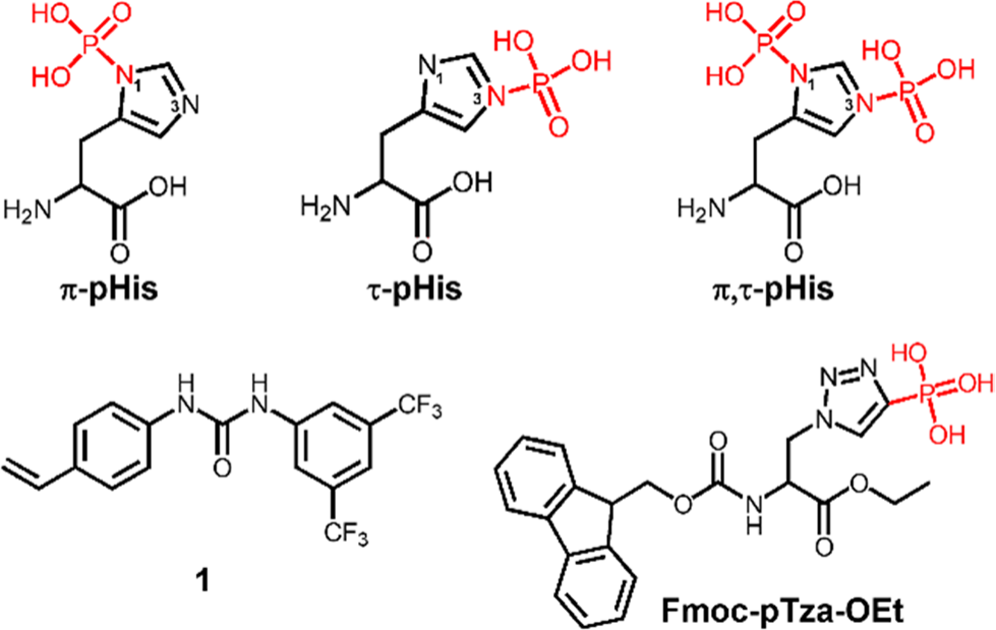
Structures of pHis isomers, neutral-urea-based functional monomer **1**, and template Fmoc-pTza-OEt.

Phosphohistidine can exist in two isomeric forms, i.e., π-pHis and τ-pHis, and the doubly phosphorylated τ, π-pHis (Figure 1), of which the former two exist in vivo.^8^ It has been estimated that histidine phosphorylation in eukaryotes accounts for 6% of the total protein phosphorylation^9^ and is more abundant than the phosphotyrosine (pTyr) modification. Nonetheless, little is known about the biological roles of pHis as compared to phosphoester modifications, i.e., phosphoserine (pSer), phosphothreonine (pThr), and phosphotyrosine. This is due to significant technical challenges in the enrichment and detection of pHis in complex biological samples. Unlike the phosphoester amino acids, the phosphoryl group of pHis is attached to a nitrogen atom, making it a phosphoramidate.

The high-energy phosphoramidate bond is susceptible to hydrolysis in acidic environments and pHis exhibits a unique stability profile.^10^ While O-phosphohydroxy amino acids (pSer, pThr, pTyr) are stable in acidic media and labile in alkali environment (pSer, pThr), the phosphoramidates display an inverse stability profile, i.e., they are base-stable and acid-labile.^11^ Most of the proteomics methods used for phosphopeptide (p-peptide) enrichment and detection involve acidic conditions during sample preparation and thus fail to preserve the pHis modification. Therefore, dedicated bioanalytical methods and protocols are needed to study pHis in proteins. Several techniques for the enrichment, detection, and analysis of pHis modification have been reported to date and reviewed elsewhere.^8,12−14^ Mild enrichment methods, ideally avoiding acidic treatment of the sample, are of critical importance for the development of pHis detection methods. Metal oxide affinity chromatography (MOAC, including TiO_2_) and immobilized metal ion affinity chromatography (IMAC), which are commonly used for enrichment of phosphorylated peptides, are in principle not suitable for enrichment of pHis since they employ acidic conditions.^15^ Nevertheless, an attempt to enrich pHis peptides using Cu^2+^-IMAC with mild acidic conditions and Fe^3+^-IMAC has been reported before.^16−20^ Recent efforts to produce phosphohistidine antibodies proved successful owing to the development of stable phosphohistidine analogues.^21−24^ Notwithstanding these advances, the drawbacks of antibodies include high production cost, poor reproducibility, and limited stability.

We report on a method for enrichment of pHis peptides using custom-made molecularly imprinted polymers (MIPs). In this proof-of-concept study, we demonstrate two approaches for selective enrichment of pHis peptides. In the first chemo-affinity approach, a pHis-MIP (MIP1) is used to enrich pHis peptides with moderate pHis discrimination. Subsequently, base-labile phosphoserine (pSer) and phosphothreonine (pThr) peptides are depleted from the sample by treatment with Ba(OH)_2_, resulting in a method obviating any acid-based sample treatment. The second method is based on new-generation pHis-MIPs (MIP2 and MIP3), which was fine-tuned in two steps with respect to binding site design and pore size distribution. The optimized pHis-MIP (MIP3) enabled enrichment of pHis peptides from tryptic digests of protein mixtures prior to analysis and sequencing by liquid chromatography–mass spectrometry (LC–MS).

## Experimental Section

### Preparation of pHis-Imprinted Polymer Particles by Crushing and Sieving

Fmoc-pTza-OEt (see Figures S1 and 1) (0.04 mmol) was dispersed in dry MeCN (0.43 mL) in a 2 mL screw-capped reaction vial equipped with a rubber septum. This was followed by addition of 1,2,2,6,6-pentamethylpiperidine (PMP, 0.08 mmol) (MIP1/NIP1) or tetrabutylammonium hydroxide (TBA·OH) (MIP2/NIP2), functional monomer **1** (Figure 1) (0.08 mmol), acrylamide (0.08 mmol), pentaerythritol triacrylate (PETA, 1.09 mmol), and finally the thermal initiator ABDV (1% w/w of total monomer mass). The solution was cooled to 0 °C and purged with a flow of nitrogen gas for 15 min, and the polymerization was initiated by placing the reaction vessel in a water bath heated to 50 °C. After 24 h, the polymer was removed from the vessel, lightly crushed, and washed with MeOH/0.1 M HCI (60:40 v/v) for template removal. The non-imprinted polymer (NIP) was prepared in the same way but in the absence of template molecule. The resulting polymers were further crushed and sieved to isolate a grain size fraction of 25–50 μm. This fraction was used for all further tests.

### Preparation of pHis-Imprinted Polymer Microspheres

Polymer microspheres (MIP3/NIP3) were prepared as reported previously^32^ by templated synthesis using AcNH@Si (1.0 g) as sacrificial template. In brief, the particles were firstly deaerated in a 50 mL Schlenk tube by three freeze–thaw cycles with intermittent N_2_-purging and then allowed to soak in prepolymerization mixtures, identical to those used to prepare MIP2 and NIP2, under continuous nitrogen flow. The volume prepolymerization mixture was adjusted to barely fill the internal pore system while avoiding particle aggregation. The mixtures were placed in a water bath and heated to 50 °C overnight. The resulting composite beads were treated in 3 M NH_4_HF_2_ solution to remove the silica template, followed by pHis-template removal as described in the previous section.

### Template Binding Tests

Each polymer (10 mg) was suspended in 1.0 mL of an equimolar mixture of Fmoc-pTza-OEt, Fmoc-pTyr-OEt, and Fmoc-pSer-OEt (each 100 μM) prepared in MeCN/H_2_O at different ratios buffered with 0.1% triethylamine (TEA). Each suspension was shaken vigorously for 2 h and then each sample was centrifuged. The supernatant (500 μL) was dried by using a Genevac EZ-2 evaporator, then reconstituted in 40% MeCN + 0.1% trifluoroacetic acid (TFA) (200 μL), and analyzed by reversed-phase high-performance liquid chromatography (HPLC). The column used was Prodigy 5 μm ODS-3 100 Å (Phenomenex, 150 × 4.6 mm^2^). Mobile phases were (A) 99.9% H_2_O + 0.1% TFA and (B) 99.9% MeCN + 0.1% TFA. A linear gradient method involving 40% B to 60% B in 12 min at a flow rate of 0.6 mL/min was used. The injection volume was 50.0 μL and the detection was performed by UV absorbance measurement at 265.0 nm. All experiments were performed in three parallel replicates.

### Synthesis of pHis Reference Peptides

MIP selectivity was probed using two different pHis peptides, VpHI and DRVYIpHPF, obtained by chemical phosphorylation of VHI and DRVYIHPF, respectively, using potassium phosphoramidate (see Supporting Information).^25^ This method is highly selective towards histidine and no other amino acid residues are phosphorylated. Furthermore, the major product of the reaction is the τ-pHis isomer. The identity of the two pHis peptides was confirmed by electrospray ionization mass spectrometry (ESI MS). The chemical shifts of the imidazole ring protons in the ^1^H nuclear magnetic resonance (NMR) spectra of VpHI peptide (Figure S2) were in agreement with previously reported data for τ-pHis isomers.^25,26^ Amino acid sequencing by matrix-assisted laser desorption ionization tandem mass spectrometry (MALDI MS/MS) of the octapeptide (Figure S3) confirmed phosphorylation of the histidine residue.

### Investigation of pHis Peptide Stability

A solution of each peptide (50 μM) prepared in 0.1% TFA, 0.1% FA, and 10 mM NH_4_HCO_3_ (pH = 8) was sampled at time points 0, 1, 2, 4, 6, 12, and 24 h followed by injection (100 μL) and analysis by HPLC. The concentration of phosphorylated peptide was determined by using external standards.

### Binding Test Using Tri-Peptide Standards

Each polymer (10 mg) was suspended in 1.0 mL of an equimolar mixture of VpSI, VpHI, VEI, and VHI (each 20 μM) prepared in MeCN/H_2_O at different ratios buffered with 0.1% TEA. After the binding test, the supernatant was dried and each sample was reconstituted using 10 mM NH_4_HCO_3_ solution (200 μL) and analyzed by reversed-phase HPLC. The column used was XBridge C18 5 μm (50 × 4.6 mm^2^). Mobile phases were (A) 10 mM NH_4_HCO_3_ and (B) MeCN/10 mM NH_4_HCO_3_ (9:1). A linear gradient method involving 0% B to 35% B in 18 min at a flow rate of 0.6 mL/min was used. The injection volume was 50.0 μL and the detection was performed by UV absorbance measurement at 265.0 nm. All experiments were performed in three replicates.

### β-Elimination Protocol

VpSI and VpTI were dissolved separately in 0.1 M Ba(OH)_2_ (500 μL) in the presence of VpHI and VHI (concentration of each peptide = 5 mM). Each solution was kept at 50 °C overnight and the percentage dephosphorylation of VpSI and VpTI was analyzed at the time points 0, 6, 12, 24, and 48 h by HPLC. At each time point, the sample was firstly neutralized with 1.0 M HCI and acidified with 0.1% FA to pH = 5. The sample was passed through a C18 silica gel column (activated with MeCN + 0.1% FA and equilibrated with 0.1% FA prior to use), the column was washed with 0.1% FA, and the peptides were eluted with 95% MeCN + 0.1% TEA. Complete dephosphorylation of all phosphoesters was confirmed after 24 h of treatment with the base media.

### Tryptic Digest of Bovine Serum Albumin (BSA)

Bovine serum albumin (BSA, 10 mg) dissolved in 100 mM NH_4_HCO_3_ (1 mL) was reduced with 50 mM dithiothreitol (DTT) for 30 min at 50 °C and alkylated with 100 mM IAA for 30 min at room temperature in the dark. Then, the protein was digested with trypsin (200 μg) for 24 h at 37 °C. The peptide sample was then acidified with TFA and desalted on a C18 silica gel column (Bond Elut C18, Analytichem International, CA). The column was first activated with MeCN + 0.1% TFA (2 mL) and conditioned with 0.1% TFA (2 × 2 mL). Finally, the peptides were eluted with 80% MeCN + 0.1% TFA (2 mL); the digest was lyophilized and stored at −20 °C prior to use.

### pHis Peptide Enrichment by Combined MIP and β-Elimination

The mixture of VpHI (40 μM), VpSI (40 μM), and tryptic digest of BSA (200 μM) (spiking level is 1:5) was prepared in 0.2 M Ba(OH)_2_ (25 μL) and equilibrated at 37 °C for 2 h. Then, 0.4 M of (NH_4_)_2_SO_4_ (12.5 μL) was added and BaSO_4_ was formed as a white precipitate. The sample was filtered to remove the insoluble BaSO_4_ salt and the residue was washed with water (12.5 μL). The filtrate was diluted with MeCN + 0.1% TEA (950 μL). This sample (200 μL) was mixed with MIP and NIP (5 mg) separately in a 1.5 mL microcentrifuge plastic tube and shaken for 2 h. After incubation, the samples were transferred to a 200 μL pipette tip microcolumn protected with a C8 plug and passed through the microcolumn. The flow-through fraction was collected as a first step of the enrichment process. The washing step was performed with 95% MeCN + 0.1% TEA (2 × 200 μL) and the elution step was performed with 50% MeCN + 0.1% TEA (2 × 200 μL). Each fraction was dried (Genevac EZ-2 evaporator), redissolved in water (200 μL), and then analyzed by HPLC with ESI-MS detection.

### HPLC ESI-MS Analysis

The HPLC ESI-MS analyses were performed in the reversed-phase mode using an analytical C18 column (XBridgeTM C18 5 μm 50 × 4.6 mm^2^, Waters, Milford, MA). The mobile phases were (A) 10 mM NH_4_HCO_3_ and (B) MeCN/NH_4_HCO_3_ (9:1). The chromatography was performed using a linear gradient from 0% B to 100% B in 30 min at a flow rate of 0.5 mL/min. The injection volume was 100 μL and the detection was performed by UV absorbance measurement at 210 nm and ESI-MS (Waters, micromassZQ, MM1, LAA1108, Milford, MA) selective ion monitoring (SIM) with the mass window of 1.0 Da. The mass spectra were recorded in positive ion mode. The analysis parameters were set as follows: capillary voltage 5 kV, cone voltage 30 V, source temperature 120 °C, and desolvation temperature 250 °C.

### LC–MS/MS Analysis of Phosphopeptides

Dried peptide samples were redissolved and analyzed on a Dionex Ultimate 3000 RSLCnano system coupled online to an Orbitrap Fusion Tribrid mass spectrometer (Thermo Scientific). To avoid loss of pHis, dried fractions were re-dissolved in cold water, vortexed for 1 min, and sonicated for 1 min immediately before LC–MS/MS analysis. The volume was adjusted so as to inject 60 fmol of peptides in a single injection of 50 μL. The injected peptide samples were desalted on-line using a C18 trap column (300 μm i.d. × 5 mm, 5 μm, 100 Å/PepMap TM 100, Thermo Scientific) for 3 min using 2% MeCN in 0.1% FA. Peptides were eluted and separated on an in-house packed 75 μm i.d. × 30 cm column with ReproSil-Pur 120 C18 1.9 μm particles (Dr. Maisch) at a flow rate of 300 nL/min with a linear gradient of 2–34% solvent B (95% MeCN + 0.1% FA) against solvent A (99.9% H_2_O + 0.1% FA).^27^ Precursor ion (MS) spectra were acquired in the *m*/*z* range 350–1800 (2+ to 4+ ion charge state) at a mass resolution setting of 120k at *m*/*z* 400 in profile mode, with an AGC target of 2e5 ions and 3 s cycle time. Precursor ions were fragmented with higher-energy collisional dissociation (HCD, NCE = 33%) and dynamically excluded for 15 s. Fragments were detected in the Orbitrap at a mass resolution setting of 15 000 at *m*/*z* 400 in centroid mode. For the analysis of the microspheres MIP3 and NIP3, MS1 mass resolution was set to 60k and precursors were fragmented with internally calibrated electron transfer dissociation (ETD) with HCD supplemental activation (EThcD). Fragment ions were analyzed in the Orbitrap at 30k mass resolution.

### Enrichment of VpHI from Tripeptides Spiked in BSA/β-Casein Digest

The three short p-peptides (VpHI, VpSI, VpTI) were combined in an equimolar mixture prior to mixing them with BSA/β-Casein digest at 1:10 and 1:20 ratio, respectively. Fifty picomoles of BSA/β-Casein were used per enrichment and were spiked with 5 pmol of each peptide for the 1:10 ratio and 2.5 pmol for the 1:20 ratio. Prior to solid-phase extraction (SPE), the peptide mixture was mixed with MeCN and 0.1% TEA to a final solvent composition of 95% MeCN + 0.1% TEA in 300 μL. Peptides were then incubated with 3 mg of MIP or NIP for 2 h with vigorous shaking in a low-binding 1.5 mL Eppendorf tube. Three replicates for each spiking level were performed on each polymer, with three pre-enrichment control samples (R, reference) for each spiking level. After the first incubation, tubes were centrifuged for 5 min at 14k rpm before the liquid was collected. Next, the MIP/NIP was washed with 400 μL of load/wash solvent for 15 min and the liquid was pooled with the previous collection and labeled “loading + washing (L+W)” fraction. Phoshopeptides were recovered from the polymers by two sequential elutions with 500 μL of elution (E) solvent (99.9% MeOH + 0.1% TEA) for 15 min and then for 30 min. The liquid fractions were pooled and labeled “E”. Both L+W and E fractions were dried by vacuum centrifugation and redissolved prior to LC–MS/MS analysis. Every fraction was injected with the same theoretical amount of tripeptide (60 fmol) by adjusting the volume. The peak areas of the tripeptides were calculated using the Genesis algorithm implemented in the LC–MS/MS data analysis software FreeStyle (v. 1.3, Thermo Scientific). Tryptic peptides from BSA/β-Casein were identified and annotated by processing the LC–MS/MS data using the MASCOT search engine (Matrix Science) through Proteome Discoverer 2.4 (Thermo Scientific).

### Chemical N-Phosphorylation of Myoglobin

Horse heart myoglobin (Mb) was chosen for chemical *N*-phosphorylation of histidines using potassium phosphoramidate (PPA). Mb contains 11 histidine residues and no reactive cysteine residues. Approximately 1 mg of myoglobin was dissolved in 1 mL of water by thorough vortexing. PPA was added to the protein solution in a 1:600 protein/PPA molar ratio and incubated at room temperature for 24 h. Phosphorylation was confirmed by intact protein mass determination using static nanoESI-MS (Orbitrap Fusion Tribrid, Thermo Scientific). Nanoelectrospray needles were obtained from Fisher Scientific (NC0355451). The protein (50 pmol) was desalted/concentrated using a C8 membrane plug in a 200 uL pipette tip and eluted in a small volume of 50% MeCN + 1% FA. Mass spectra were acquired in the Orbitrap mass analyzer at 120k resolution with a 70% RF lens, 5e5 AGC target, and 100 ms maximum injection time for a total acquisition time of 2 min.

### Enrichment of pHis Peptides from the Mixture of the Mb/BSA/β-Casein Digest

Protein digests of equimolar mixtures of BSA, β-Casein, and histidine phosphorylated myoglobin were initially de-salted using porous R3 resin. A 200 μL pipette tip was plugged with C18 membrane and filled with 750 μg of R3 resin. The beads were conditioned with 100% MeCN and equilibrated with 100 μL of water. For either MIP or NIP, a total of 7.36 μg of protein digest (200 pmol) was loaded on the tip and the liquid passed through with manual centrifugation. The flow-through was collected and passed once more through the tip. The beads were washed twice with 100 μL of water before being eluted with 50 μL of 50% MeCN. The eluted peptides were dried and redissolved in water prior to SPE. The de-salted digest was re-dissolved in 60 μL of 0.1% TEA and divided into three aliquots of 15 μL (50 pmol). Then, the aliquots were diluted with 99.9% MeCN + 0.1% TEA to a final volume of 300 μL. LC–MS/MS analysis and peptide identification were performed as described in the previous section.

## Results and Discussion

### Design and Synthesis of a First-Generation pHis MIP (MIP1)

We previously showed that imprinted polymers (MIPs) prepared using urea-based *N*-3,5-bis(trifluoromethyl)-phenyl-*N*′-4-vinylphenylurea, functional monomer **1** (Figure 1), display a high affinity for phosphorylated peptides^28,29^ and that the selectivity for either phosphoserine (pSer) or phosphotyrosine (pTyr) peptides can be programmed using the appropriate template.^30−32^ Mechanistically, the hydrogen bond-driven recognition and the charge-neutral resin character distinguish these phases from currently used phosphoenrichment tools, e.g., IMAC, TiO_2_, and antibodies, and could explain the reduced charge-dependent sequence bias of the enriched phosphopeptide pool. Here we adopted the same urea-based approach for the recognition of histidine-phosphorylated peptides.

One of the key elements in the design of the imprinted polymer is the choice of the template. The labile nature of phosphohistidine posed many problems when used as an antigen to raise antibodies since it undergoes rapid dephosphorylation after injection into animals.^10^ Inspired by previous reports on stable phosphohistidine analogues,^12,21,33^ we prepared Fmoc-pTza-OEt (Figure 1), which mimics the τ-pHis isomer, and used it as a template for imprinting. The template was synthesized according to Figure S1 using the copper-catalyzed click reaction. Imprinted polymers were then prepared by free radical polymerization using a 1:2 ratio of template (as its PMPH or TBA salt) to **1**, pentaerythritol-triacrylate (PETA) as cross-linker, and acetonitrile (MeCN) as porogen in bulk or microsphere format as described in the Supporting Information (Table S1).

### Probing Affinity and Selectivity of Polymers in Simple Amino Acid and Peptide Mixtures

Previously reported protocols for MIP-based enrichment of pTyr and pSer peptides employed acidic (0.1% TFA) loading, washing, and elution steps.^28−30,34^ Under these conditions the phosphate group is not fully protonated and with one oxygen negatively charged, it is a potent hydrogen bond acceptor that can interact with the urea N–H hydrogen bond donor. Such conditions are however not appropriate for enrichment of pHis due to its acid-labile nature. Instead, phosphoramidates such as pHis are stable under basic conditions, and since the phosphate group is deprotonated, it engages in strong interactions with the urea functional groups of the MIP. We therefore used triethylamine (TEA) as mobile phase modifier to ensure the stability of pHis modification and to provide strong interactions with the imprinted polymer.

**Figure 2:**
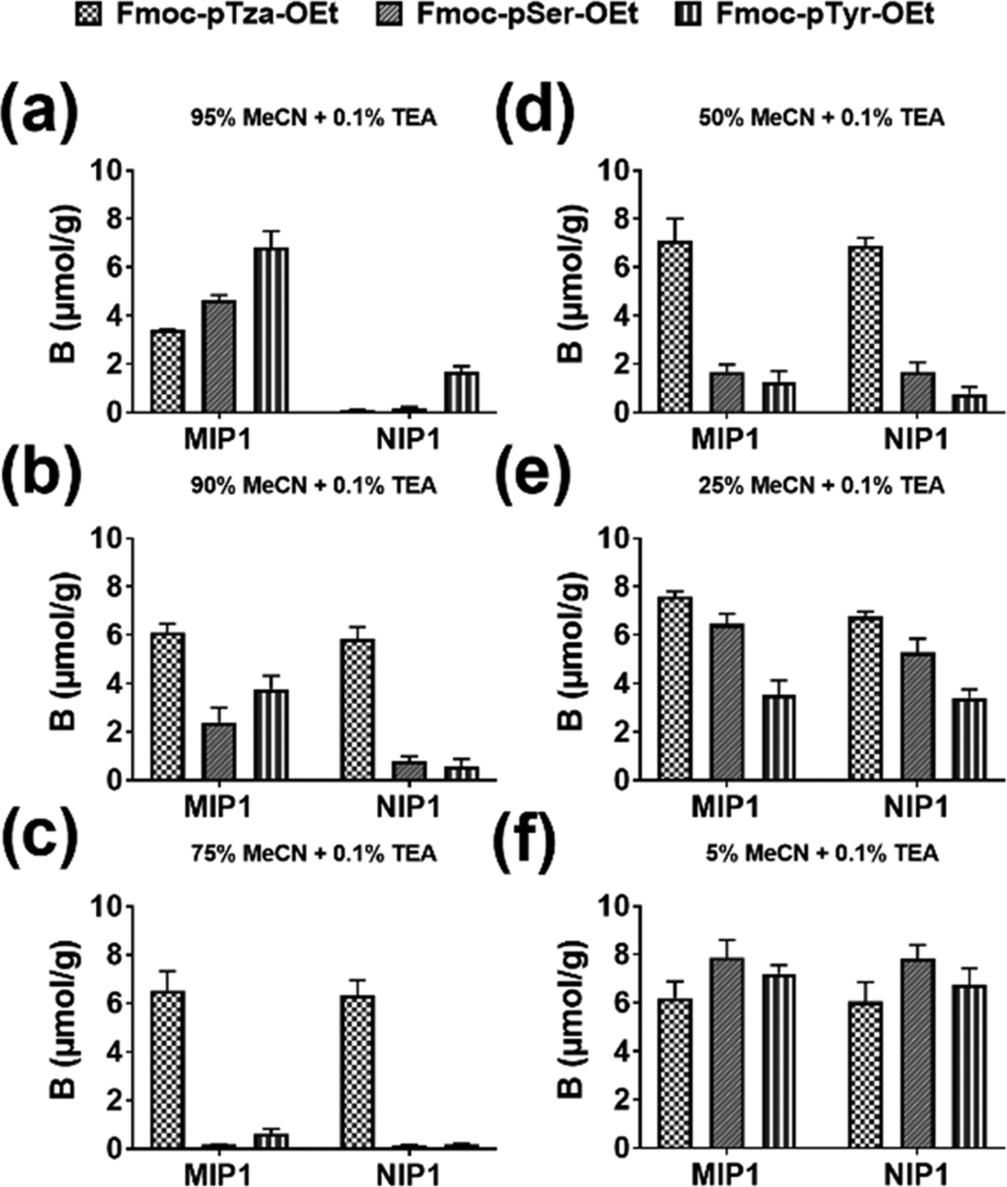
Absorbed amounts of Fmoc-pTza-OEt, Fmoc-pSer-OEt, and Fmoc-pTyr-OEt by imprinted and non-imprinted polymers in different solvent systems: (a) 95%, (b) 90%, (c) 75%, (d) 50%, (e) 25%, and (f) 5% MeCN and each buffered with 0.1% TEA.

We first tested the affinity of MIP1 and NIP1 towards N,C-protected amino acids pTza, pSer, and pTyr in MeCN/H_2_O mixtures buffered with 0.1% TEA as basic modifier (Figure 2). As seen in Figure 2a, the contrasting retention behavior of MIP1 and NIP1 in 95% MeCN reflects a strong impact of imprinting. Although the MIP captured each of the phosphorylated amino acids, the highest imprinting factor (IF = B_MIP_/B_NIP_) was registered for Fmoc-pTza-OEt (see Table S2). This reflects a slight memory for the template accompanied by a rather strong nonspecific contribution. The latter increased dramatically with further increase in the water content. Thus, retention differences between MIP1 and NIP1 could no longer be observed at 10% water and beyond. However, under these conditions both polymers showed a pronounced selectivity for the pTza template. Given that both MIP1 and NIP1 displayed this effect, we attribute this to an involvement of the triazole ring in the polymer template interactions. A plausible hydrogen-bonding motif places the triazole nitrogen juxtaposed to the phosphate group at hydrogen bond distance from the urea group (Figure S4), thereby stabilizing additionally the monomer–template complexes.

**Figure 3:**
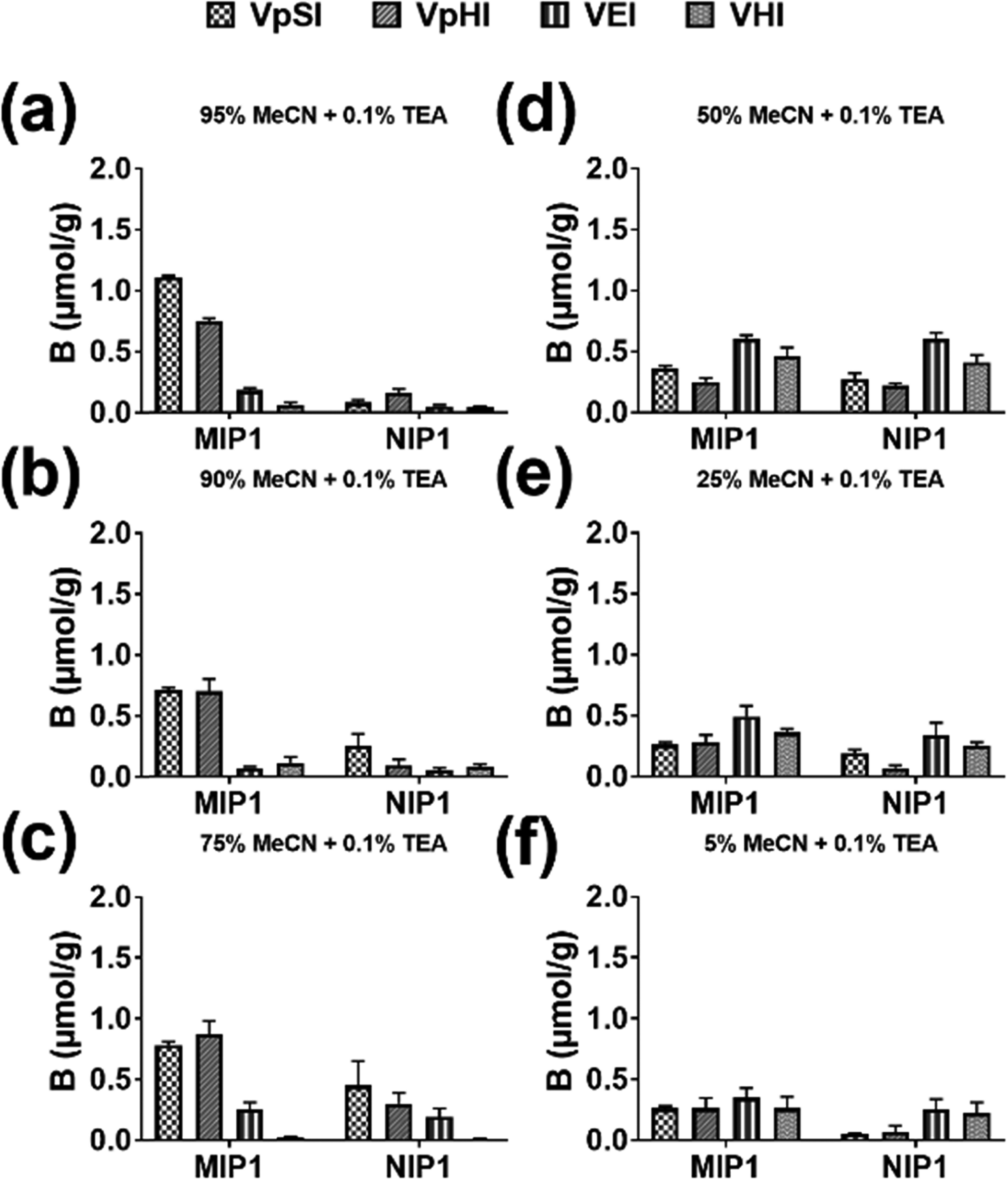
Amounts of VpSI, VpHI, VEI, and VHI bound to imprinted and non-imprinted polymers in different solvent systems: (a) 95%, (b) 90%, (c) 75%, (d) 50%, (e) 25%, and (f) 5% MeCN and each buffered with 0.1% TEA.

We next probed the ability of the imprinted polymer to recognize the histidine phosphorylated peptide VpHI in the presence of structurally related VHI, VpSI, and VEI in MeCN/H_2_O mixtures + 0.1%TEA as modifier (Figure 3). Also, in this case we observed the strongest imprinting effect in 95% MeCN, reflected in a high binding on the MIP1 and low binding on the NIP1 (see IF values, Table S3). Moreover, despite a clear preference for the phosphopeptides VpSI and VpHI over the negatively charged VEI, no significant pHis selectivity was noted in this test. Instead, we noted an overall decrease in both binding and imprinting effect with increasing water content. The absence of pHis selectivity further supports the above-invoked role of the triazole nitrogen in the urea template interactions (vide supra).

**Figure 4:**
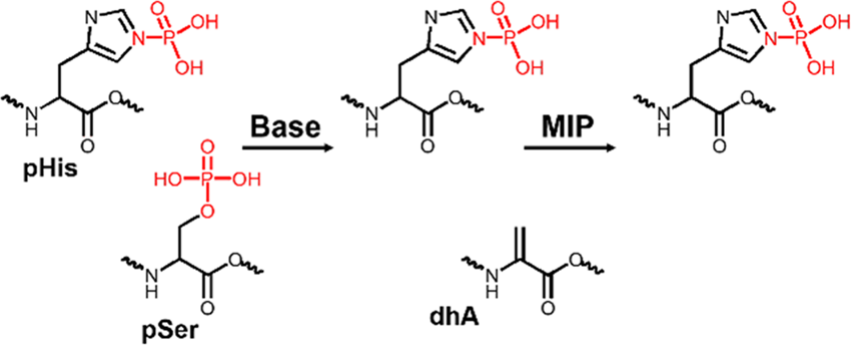
Concept of the MIP-based method for enrichment of pHis peptides. pSer (or pThr) undergoes facile dephosphorylation via β-elimination in the presence of a strong base (e.g., sodium or barium hydroxide) and it is converted to dehydroalanine (dhA). pHis stability is not affected under such conditions and it can be enriched by the MIP in the next step.

Considering the lack of strong pHis discrimination, we turned to an alternative enrichment approach where pSer and pThr peptides were selectively dephosphorylated in the presence of pHis peptides followed by the MIP-based enrichment step (Figure 4).

### pHis Discrimination by β-Elimination of pSer and pThr

Phosphohistidine modification is known to undergo facile dephosphorylation in acidic environment. The synthesized VpHI and DRVYIpHPF were therefore subjected to a stability test (Figure S5). Indeed, the studied peptides were dephosphorylated in the presence of acids in contrast to basic buffer, where no significant decomposition was observed even after 24 h. However, a short treatment (<30 min) of the peptides with acids did not lead to extensive dephosphorylation. Using short acidic LC gradients is another approach for detection of pHis by LC–MS/MS.^14,19^ In another study, copper-immobilized metal ion affinity chromatography (Cu^2+^-IMAC) was adopted for extraction of pHis peptide from a digest of phosphorylated HPr protein from *Escherichia coli*.^16^ The sample was in this case loaded on a column conditioned with 0.1% acetic acid and the washing step was performed with 0.1% acetic acid in 50% MeCN. However, the stability of individual pHis peptides can vary depending on the sequence and thus ideally any acidic treatment should be avoided.

We therefore took advantage of the contrasting stability profile of pHis and pSer/pThr in alkaline environment (Figure 4). This approach was used to distinguish these modifications in a histidine kinase assay.^35,36^ Both pSer and pThr undergo facile dephosphorylation via β-elimination in a strongly alkaline environment. This reaction combined with subsequent Michael addition was used as a chemical derivatization method in protocols for enrichment of pSer and pThr peptides.^37,38^ Treatment of the mixture of VpSI and VpHI with 1 M NaOH at 60 °C for 30 min led to complete dephosphorylation of VpSI to give VdhAI (dhA = dehydroalanine). VpHI was not affected under these conditions. The sample was used in a binding equilibrium test (see Supporting Information for experimental). It was clear that VdhAI did not bind to the MIP1 and thus only VpHI was captured by the imprinted polymer (Figure S6). This result verifies the advantage of β-elimination for specific capture of phosphoamidates.

### Probing Affinity and Selectivity with Spiked BSA Digest

After we confirmed the affinity of the MIP1 for the histidine phosphorylated peptide in a simple model system, we turned to more complex samples. As an initial test, we spiked 200 pmol of VpHI peptide in 1 nmol of BSA tryptic digest and incubated this mixture with MIP1 followed by transfer of the suspension to a micro-column. Next, washing and elution steps were performed as described in the Experimental Section.

**Figure 5:**
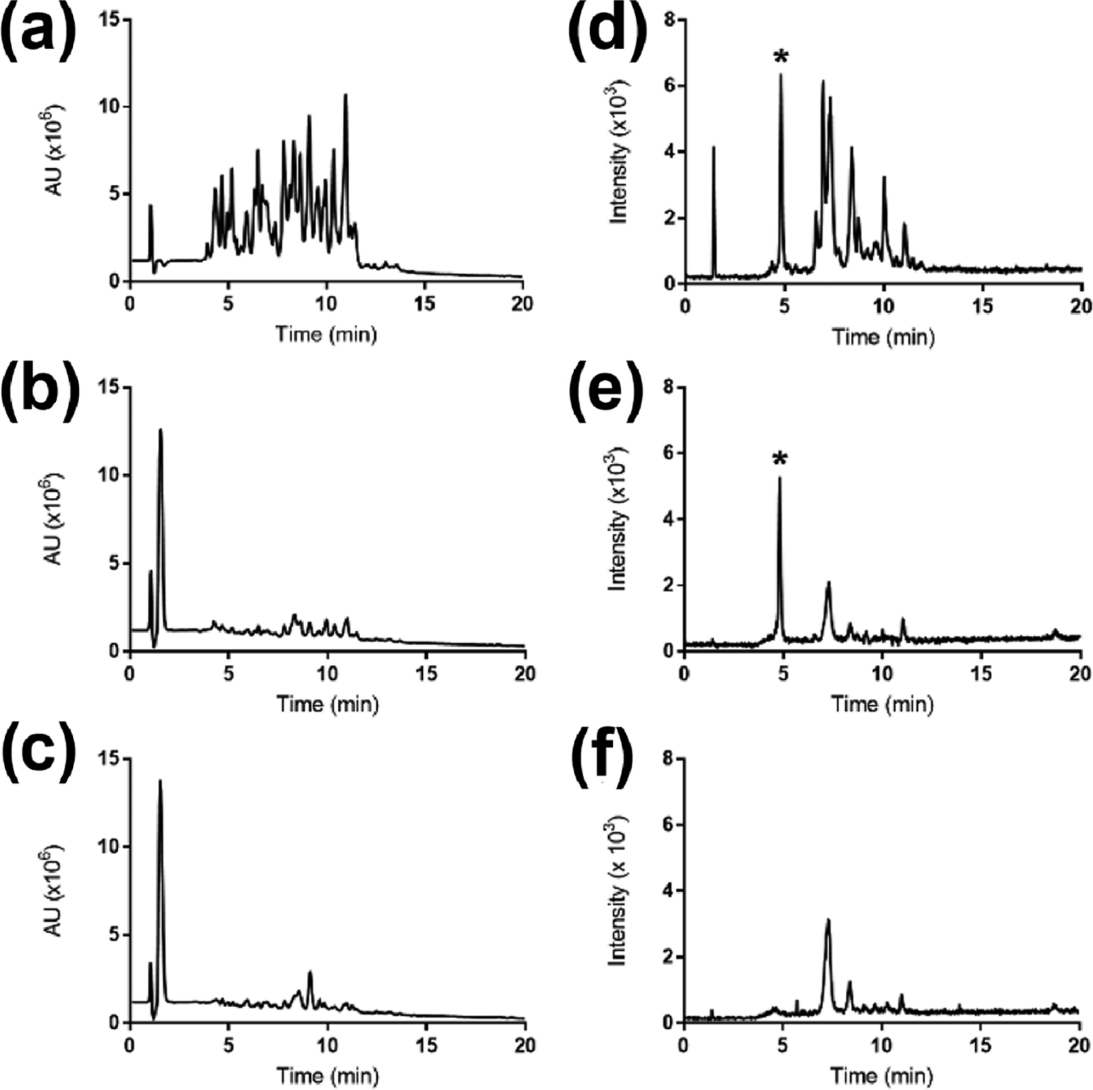
HPLC-UV chromatograms (a–c) and corresponding ESI-MS selective ion monitoring (SIM, *m*/*z* = 447.5) spectra (d–f) of the sample before enrichment (a, d) and elution fractions from MIP1 (b, e) and NIP1 (c, f). The VpHI peptide (0.2 μmol) was spiked in BSA (1 μmol) digest. The signal from VpHI is marked with an asterisk.

The original sample, the combined flow-through and washing fractions, and elution fractions from MIP1 and NIP1 were analyzed by LC ESI MS with selective ion monitoring (SIM) set at *m*/*z* 447.5 corresponding to the mass of VpHI peptide (Figures 5 and S7). Treatment of the sample with both MIP1 and NIP1 led to a significant sample cleanup as can be seen from the UV chromatograms of the elution fractions (Figure 5b,c).

However, the pHis peptide was found only in the elution fraction from the MIP1 (Figure 5e). The NIP1 on the other hand did not retain this peptide, as it was present only in the combined flow-through and washing fraction (Figure S7d).

Repeating the test using DRVYIpHPF as pHis model peptide gave a similar result and thus a significant sample cleanup (Figure S8). These results confirmed the ability of the MIP1 to recognize pHis peptides in a protein digest.

### Probing Affinity and Selectivity with Base Treated Tryptic Peptides

The dominating phosphorylated residues in proteins are pSer and pThr. The high abundance of these motifs can mask less abundant motifs such as pTyr and especially labile motifs such as pHis. Focusing on the latter, base-stable phosphorylation concept, we developed a protocol where the affinity for pHis was enhanced by elimination of the interference of the most abundant base-labile phosphorylations (pSer and pThr).

Although sodium hydroxide has been frequently used in the past for β-elimination of p-peptides, the inherent disadvantage of this approach is the sample desalting step, which requires chromatographic purification and thus generates a risk of sample loss.^39−41^ To minimize sample handling, we chose barium hydroxide as the alkaline agent. Ba(OH)_2_ can be removed from the sample by precipitation in a form of water-insoluble barium sulfate and simple filtration.^42^ Furthermore, it is more efficient in β-elimination and can be used at lower concentration (0.1–0.2 M compared to 1 M for NaOH), thus minimizing the degradation of peptides (Figure S9). Both VpSI and VpTI peptides dephosphorylated after treatment with 0.1 M Ba(OH)_2_ in 24 h. The samples were further tested with MIP1 and NIP1. It was shown that the dephosphorylated forms of pSer and pThr tri-peptides, VdhAI and VmdhAI (mdhA, β-methyldehydroalanine), respectively, did not bind to the MIP and only VpHI was captured with enhanced binding percentage (Figure S9c). This result confirms that combining the β-elimination of phosphoesters (pSer and pThr) with MIP-based enrichment enhances the specific capture of phosphoamidates (pHis).

As a proof of concept we used a sample of tryptically digested BSA spiked with both VpSI and VpHI at 1:5 spiking level. The sample was treated with barium hydroxide in order to deplete the pSer peptide.

**Figure 6:**
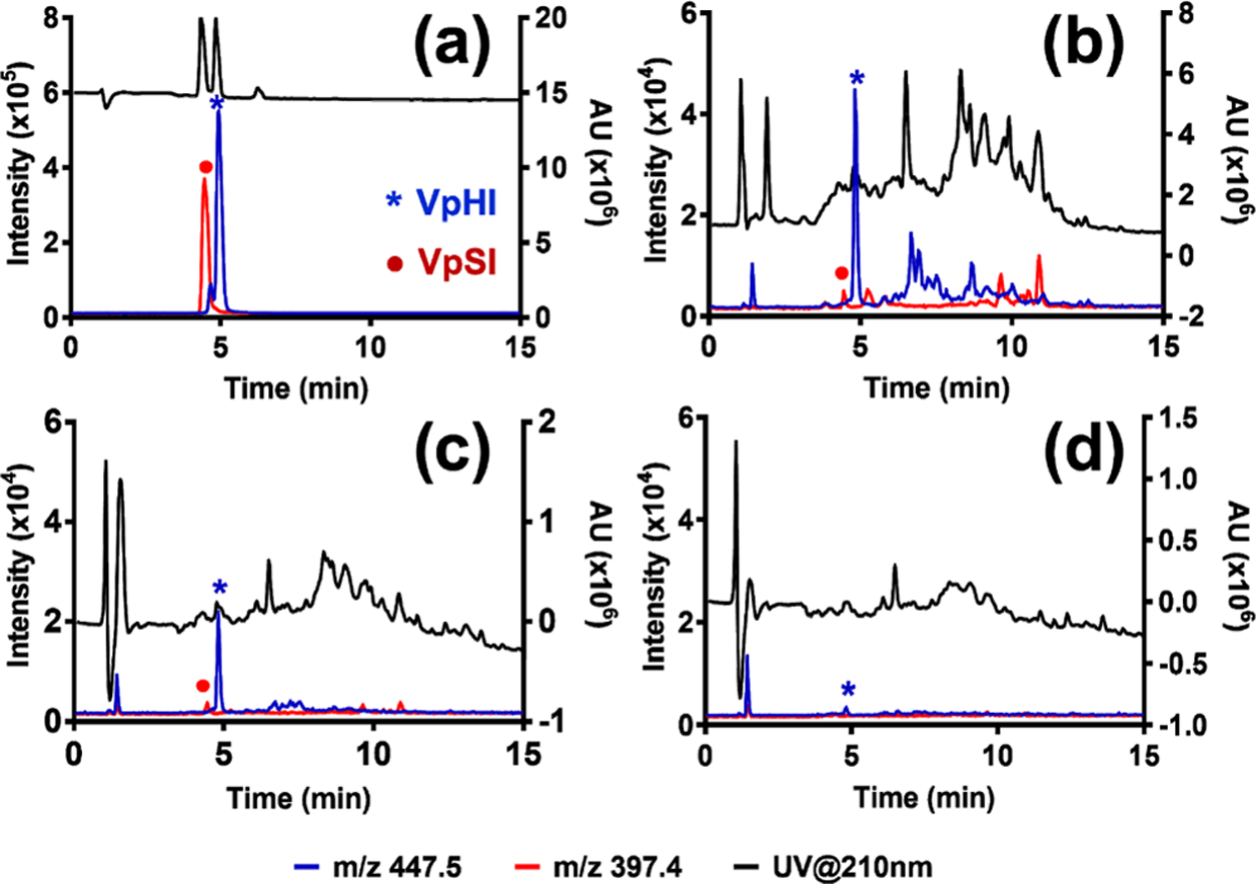
HPLC-UV chromatogram and corresponding ESI-MS selective ion monitoring of VpHI (*m*/*z* = 447.5) and VpSI (*m*/*z* = 397.4) (a), spiked in BSA tryptic digest and treated with Ba(OH)_2_ (b), and in the elution fractions from MIP1 (c) and NIP1 (d).

The samples both before and after treatment with MIP1 and NIP1 were analyzed by LC–MS (Figures 6 and S10) using SIM of ions with *m*/*z* = 447.5 (VpHI) and *m*/*z* = 397.4 (VpSI). Compared to the reference sample with only VpSI and VpHI (Figure 6a), it was clear that treatment of the spiked sample with Ba(OH)_2_ resulted in dephosphorylation of VpSI (Figure 6b). This sample was then treated with MIP1 or NIP1; only the MIP1 extraction resulted in significant enrichment of the pHis peptide (Figure 6c,d). The HPLC-UV chromatograms confirmed that a significant cleanup had been achieved.

Unfortunately, testing this method at lower spiking levels and with more complex digests led to low recoveries and reduced selectivity (Figure S11). We therefore turned to a different strategy for further optimization of the pHis-MIP affinity and selectivity.

### Design and Synthesis of a Second Generation of pHis-MIP (MIP2)

Comprehensive optimization of imprinted polymer performance involves both structural and compositional parameters. In case of the hydrogen bond-mediated imprinting of phosphoanions using urea monomers, the binding affinity and selectivity are influenced by both monomer and template structural parameters. This was covered in depth in our previous report.^43^ The nature of the counter-cation has been shown to strongly influence the template-monomer complexation and in turn the target affinity exerted by the MIP. Enhanced complex stability is obtained by the use of quaternary ammonium ions like tetrabutylammonium (TBA), also accompanied by pronounced counterion memory effects.^44^

Focusing on this parameter, we prepared MIP2/NIP2 (Table S1) in a similar manner to MIP1/NIP1 but by replacing the tertiary amine PMP with the quaternary ammonium base TBA·OH. The polymers were freed from template and processed in an otherwise identical manner. The polymers were compared by testing their ability to enrich the model pHis peptide target (VpHI) from structurally similar VpSI and VpTI tripeptides at four spiking levels of BSA/β-Casein digests (Figure S12a).

**Figure 7:**
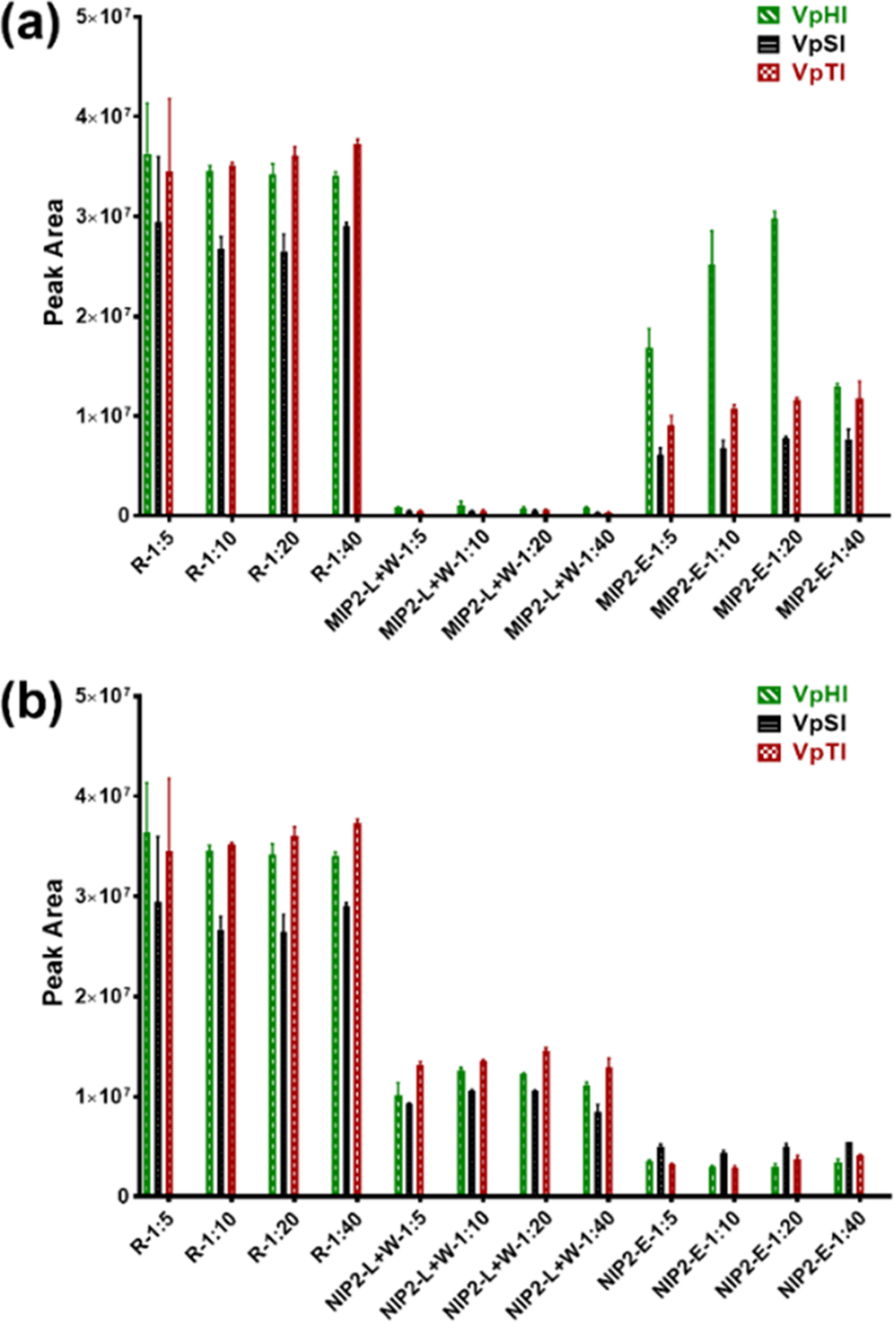
Peak areas of the three phosphorylated tripeptides obtained from each SPE fractions obtained in MIP2 (a) and NIP2 (b) at 1:5, 1:10, 1:20, and 1:40 spiking levels (R = reference, L+W = loading + washing, E = pooled elution).

We reasoned that pHis selectivity in this case would provide unequivocal evidence for the presence of side-chain-discriminative binding sites. With most of the spiked peptides recovered in the load/wash fractions, both NIPs failed to enrich any of the targets regardless of spiking level (Figures S13, S14, and 7b). MIP1 prepared using tertiary amine counterion (PMPH^+^) revealed a similar behavior (Figure S13a). On the other hand, MIP2 increased VpHI abundance relative to the other two tripeptides (Figure 7a) especially at spiking ratios 1:10 and 1:20, whereas the effect was lost at 1:40. The possible reasons for this behavior are discussed below. To further confirm this observation, the experiment was repeated in triplicate at spiking levels 1:10 and 1:20 (Figure S14).

**Figure 8:**
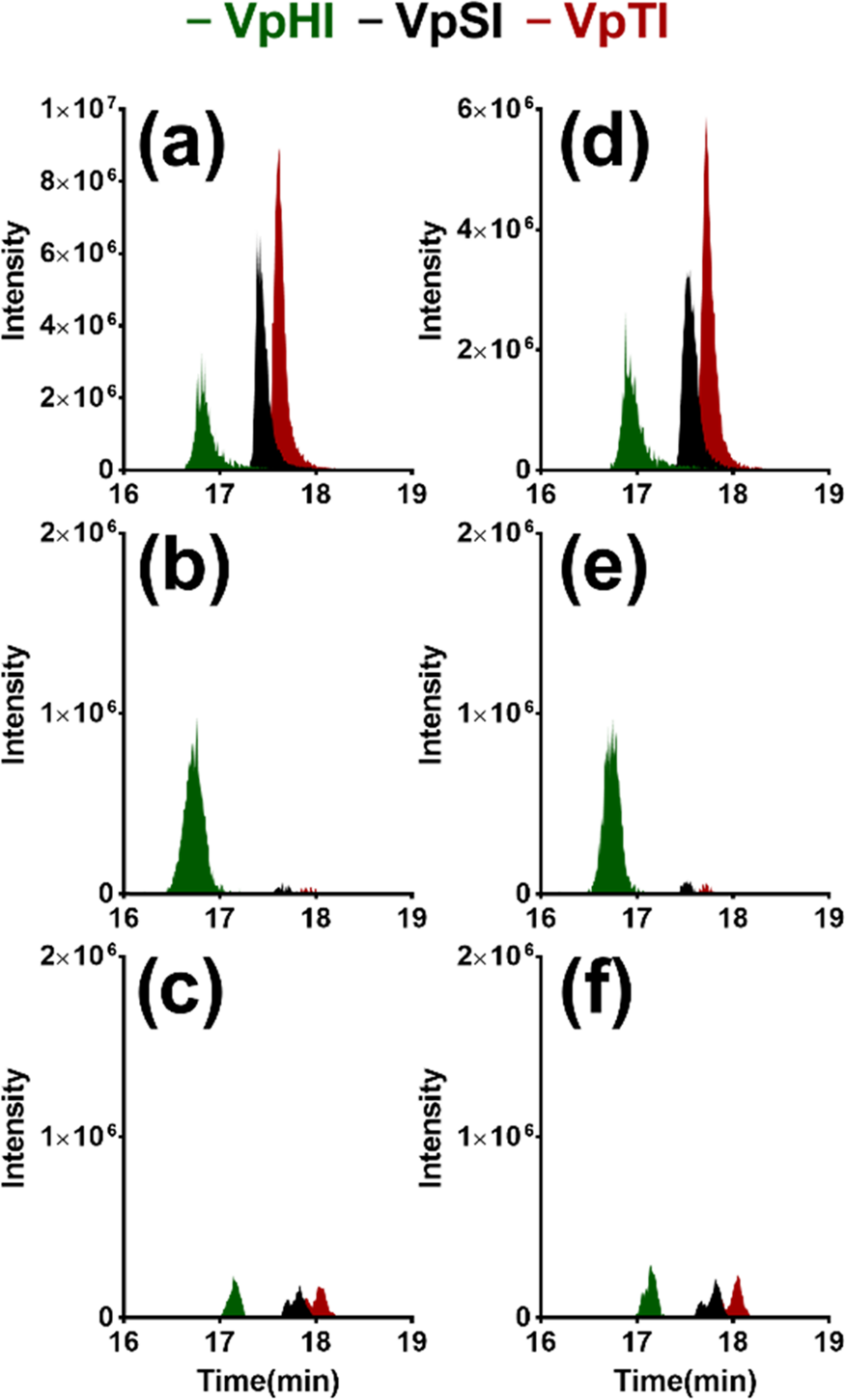
Extracted chromatogram of tripeptides (VpHI (green), VpSI (black), and VpTI (red)) for 1:10 (a–c) and 1:20 (d–f) spiking level from pre-enrichment (a, d), elution fractions obtained from MIP2 (b, e), and those obtained from NIP2 (c, f).

Here, MIP2 displayed pronounced target selectivity as seen by the relative peak area ratio (ApHis/(ApSer + ApThr)) exceeding 10 of the spiked peptides in the eluted fractions (Figure 8). The results showed that the choice of counterion, in addition to the template and urea host monomer, plays a crucial role in the generation of high-affinity sites compatible with complex matrices.

### LC–MS Test of pHis Peptide Enrichment Using MIP2

To test whether MIP2 and NIP2 were suitable for enrichment of tryptic pHis peptides from a more complex protein sample, we generated a tryptic digest of histidine phosphorylated myoglobin, BSA, and β-Casein. The enrichment protocol is shown in Figure S12b and detailed in the Experimental Section. Despite the promising results observed for the model tripeptides, the results for the endogenous tryptic peptides were not as expected, as judged by the number of phosphopeptides identified (Figure S15) and pHis peptide abundance (Figure S16). These results may have several causes. We previously ascribed the relatively low phosphoenrichment observed for complex digests to the hydrophobic character of the MIP scaffold resulting in rapid fouling and blockage of access to the discriminative sites.

Moreover, the polymers in this study were amorphous resins with a wide distribution of pores ranging from micro-pores with limited peptide access to accessible meso- or macro-pores. This may result in a size-dependent binding preference and poor accessibility for larger-sized peptides.

### Enrichment of Tryptic pHis Peptides Using Controlled-Pore-Size-Imprinted Polymers (MIP3)

We previously introduced hierarchically phosphopeptide epitope-imprinted polymers in a microsphere format to improve the overall binding efficiency and minimize the biomolecule size bias.^32^ The resulting mesoporous microspheres (MIP3) featured a controlled and narrow pore size distribution and were capable of enriching larger p-peptide fragments.

**Figure 9:**
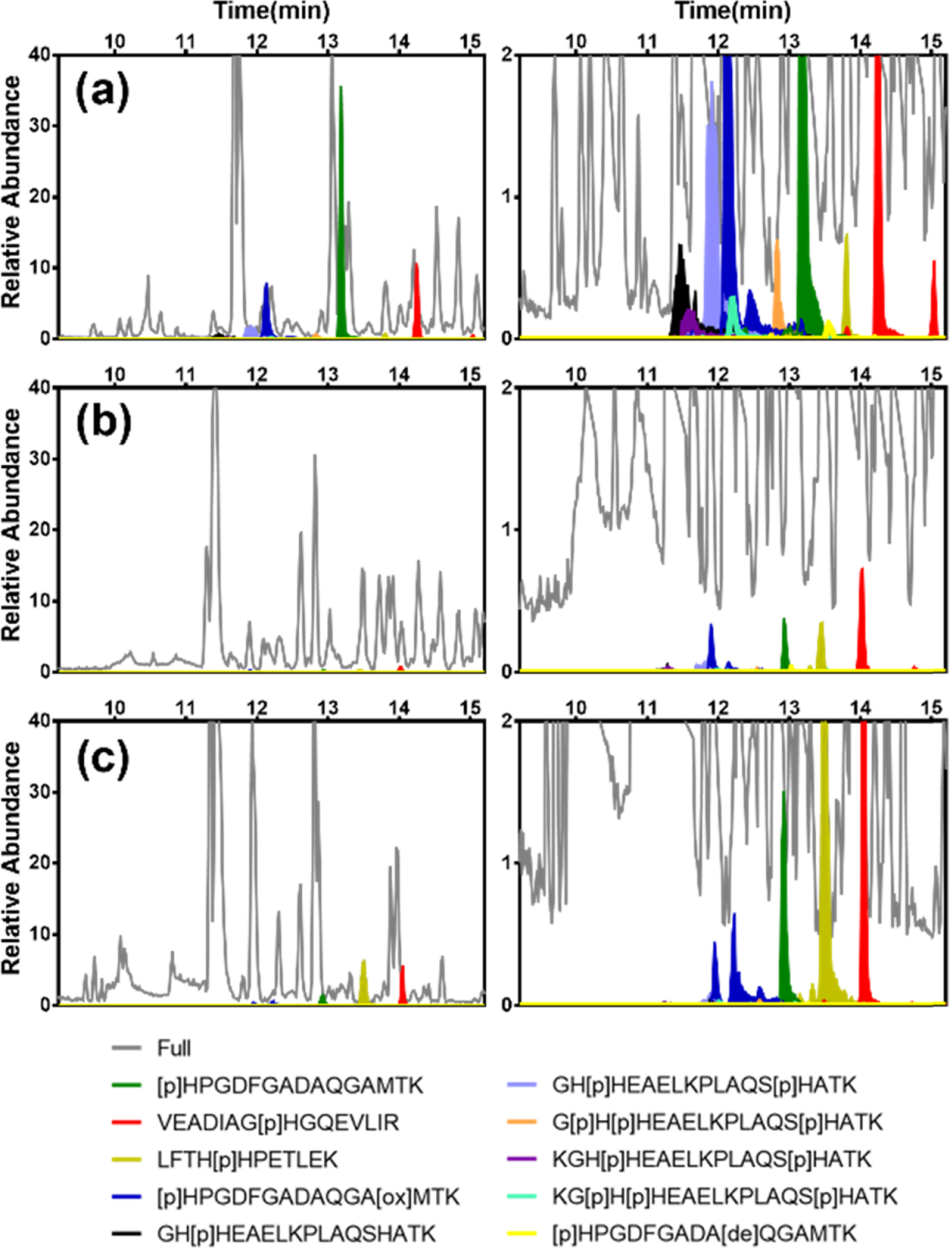
Extracted chromatograms highlighting histidine-phosphorylated peptides (indicated in color) corresponding to the elution fractions after enrichment using (a) MIP3 and (b) NIP3, and (c) no enrichment of a 1:1:1 BSA/β-Casein/myoglobin digest (the range for relative abundance is from 0 to 40 on the left and from 0 to 2 in the right).

To test the approach, we prepared pHis microspheres MIP3 and NIP3 (Table S1) and tested them on the His phosphorylated myoglobin, BSA, and β-Casein digest according to the protocol in Figure S12b. Figures 9 and S17 show the extracted chromatograms of the pre-enrichment control sample and post-enrichment (elution) sample using the two mesoporous capture materials with pHis peptides indicated in color. The latter comprise 10 mono- and multiple-His-phosphorylated peptides ranging from 11 to 18 amino acids in length (Table S4).

First, we noted a striking reduction of the matrix-related peaks (shown in gray) after MIP enrichment, most notably for retention times exceeding 15 min (Figure S17). All modified peptides were identified in the MIP3 eluate (Figure 9a and Table S4), whereas the NIP3 revealed only modified peptides of low abundance (Figure 9b). Equally striking was the ability of the MIP3 to capture multiply phosphorylated peptides (light blue, orange, purple, and turquoise chromatograms). Confident phosphorylation site localization was achieved by tandem mass spectrometry using EThcD fragmentation, producing high amino acid sequence coverage and conserving the PTM on fragment ions (Figure S18).

The number of peptide spectrum matches (PSMs) of pHis peptides (Table S4 and Figure S19) confirmed the high selectivity of MIP3 for pHis-containing peptides. We observed a strong increase in the number of pHis peptide PSMs relative to other phosphopeptides (pSer, pThr), also when compared to the pre-enrichment control sample and the eluate of NIP3. We therefore conclude that a dual MIP templating strategy and microsphere format can be used to achieve enrichment of phosphohistidine-containing peptides obtained by tryptic digestion of phosphoproteins.

## Conclusions

Protein histidine phosphorylation was reported more than 50 years ago but its biological role is still poorly understood. For many years, the acid-labile nature of pHis hampered development of methods for detection and analysis of this important PTM. This is now changing as new bioanalytical approaches and efficient tools are emerging. The molecularly imprinted polymer is such a tool that enables enrichment of pHis peptides under mild pH conditions. Using a refined pHis selective capture phase with tailored porosity and binding chemistry we have demonstrated a method, MIP3, offering the possibility to extract pHis peptides from complex mixtures under such mild conditions. This now opens new possibilities for highly robust modification-specific peptide enrichment strategies that we will continue to explore. We foresee that MIP-based methods for enrichment of pHis will play an important role in unraveling the biological roles of phosphohistidine in proteins using large-scale proteomics approaches.

## Supporting Information Available

Detailed overview of experimental section outlining synthesis of template, reference peptide, and supporting tables and graphs (PDF)
