# Supplementary material for: Selective Enrichment of Histidine Phosphorylated Peptides Using Molecularly Imprinted Polymers
Source: Anal Chem. 2021 Feb 16;93(8):3857–66. doi: 10.1021/acs.analchem.0c04474 (PMC8023515; doi:10.1021/acs.analchem.0c04474)
Supplement: Supplementary file 1 — ac0c04474_si_001.pdf [file ac0c04474_si_001.pdf]

Supporting Information

Selective enrichment of histidine phosphorylated peptides using molecularly imprinted polymers

Anıl Incel[†], Ignacio Arribas Díez[‡], Celina Wierzbicka[†], Katarzyna Gajoch[†],

Ole N. Jensen[‡] and Börje Sellergren[*,†]

[†]Department of Biomedical Science, Faculty of Health and Society, Malmö University, 205 06 Malmö, Sweden

[‡]Department of Biochemistry & Molecular Biology and VILLUM Center for Bioanalytical Sciences, University of Southern Denmark, DK-5230 Odense M, Denmark

Contents

Page – S-4: Experimental

Page – S-4: Materials

Page – S-5: Apparatus and methods

Page – S-5: Synthesis of Fmoc-pTza-OEt

Page – S-7: Synthesis of pHis reference peptides

Page – S-8: Tables

Page – S-8: Table S1: Prepolymerization solution compositions and formats used to prepare imprinted and non-imprinted polymers

Page – S-9: Table S2: Imprinting Factors (IF) for Fmoc-protected templates corresponding to the data in Fig.

Page – S-9: Table S3: Imprinting Factor (IF) for tri-peptides corresponding to the data in Fig. 3

Page – S-10: Table S4: List of identified phosphohistidine peptides

Page – S-11: Figures

Page – S-11: Figure S1: Synthesis of pHis analogue template Fmoc-pTza-OEt (**2**). a) Fmoc-OSu, $NaHCO_3$, acetone/water; b) DEAD, PPh_3, HN_3, THF; c) diethyl ethynylphosphonate, $CuSO_4$ x 5 H_2O, sodium ascorbate, tBuOH/water; d) 1. TMSBr, DCM, 2. MeOH.

Page – S-11: Figure S2: Portion of NMR spectra (6.5-8.0 ppm) of (a) VHI and (b) VpHI (partially dephosphorylated) in D_2O at pH 10.

Page – S-12: Figure S3: MALDI MS/MS spectra of DRVYIpHPF peptide (precursor ion *m/z* = 1126.505)

Page – S-12: Figure S4: Hydrogen bonding association of urea monomer, **1** with pTza-based template (a) and pHis peptide (b).

Page – S-13: Figure S5: Stability profile of (a) VpHI and (b) DRVYIpHPF in 0.1% TFA, 0.1% FA and 10mM NH_4HCO_3 (pH=8).

Page – S-14: Figure S6: HPLC-UV chromatograms from a binding equilibrium test of an equimolar mixture of (a) VpHI and VpSI, (b) VpHI and VdhAI (resulting from dephosphorylation of VpSI) in 95% ACN + 0.1% TEA in presence of MIP1 and NIP1. The bars show the average of three replicas and the error bars represent the corresponding standard deviation.

Page – S-15: Figure S7: HPLC-UV chromatograms (a, b) and corresponding ESI-MS selective ion monitoring (SIM, m/z=447.5) spectra (c, d) of the flow through + washing fractions from MIP1 (a, c) and NIP1 (b, d) after loading a sample of digested BSA (1 µmol) spiked with VpHI (0.2 µmol). The signal from VpHI is marked with an asterisk.

Page – S-16: Figure S8: HPLC-UV chromatograms (a-e) and corresponding ESI-MS selective ion monitoring (SIM, m/z=1126.5) spectra (f-j) of a sample of digested BSA (1 µmol) spiked.with DRVYIpHPF (0.2 µmol) before enrichment (a, f), and the flow through + washing fractions from MIP1 (b, g) and NIP1 (c, h) and elution fractions from MIP1 (d, i) and NIP1 (e, j) after solid phase extraction. The signal from DRVYIpHPF is marked with an asterisk.

Page – S-17: Figure S9: HPLC-UV chromatogram of p-peptide mixtures containing VpSI (a) and VpTI (b) before and after β–elimination (0.1 M $Ba(OH)_2$ @50 ºC for 24 h). VpSI and VpTI=1, VpHI=2, VHI=3, VdhAI and VmdhAI=4. Binding degree of VdhAI (left) and VmdhAI (right) in the presence of VpHI and VHI on MIP1 and NIP1 in 95% MeCN + 0.1% TEA (c).

Page – S-18: Figure S10: HPLC-UV chromatograms of a sample of digested BSA (1 µmol) spiked with VpSI and VpHI (0.2 µmol each) and treated with $Ba(OH)_2$ (a), the combined flow through and washing fractions from MIP1 (b) and NIP1 (c), a mixture of VpSI and VpHI (d) and the elution fraction from MIP1 (e) and NIP1 (f) and the BSA digest with spiked peptides before beta-elimination (g). The signals from VpSI and VpHI are marked with a dot and asterisk, respectively.

Page – S-19: Figure S11: Peak areas of the three phosphorylated tripeptides obtained from each SPE fraction in MIP1 and NIP1 fractions of 1:10 spiking level. (R=pre-enrichment, L+W=loading+washing, E=pooled elution)

Page – S-20: Figure S12: Schematic representation of the workflows. a) Three short phosphopeptides were first spiked in BSA+ B-Casein digest in two spiking levels (1:10 and 1:20) before SPE. Load+Wash and Elution fractions were collected and dried prior LC-MSMS analysis. b) Protein digests were first de-salted and dried prior MIP SPE. Load+Wash and Elution fractions were collected and dried prior LC-MS/MS analysis.

Page – S-21: Figure S13: Peak areas of the three phosphorylated tripeptides obtained from each SPE fractions obtained in MIP1 (a) and NIP1 (b) at 1:10 and 1:20 spiking level (R=reference, L+W=loading+washing, E=pooled elution).

Page – S-22: Figure S14: The extracted chromatogram of tripeptides (VpHI, VpTI, and VpSI) for 1:10 spiking level (a) and 1:20 spiking level (b) of pre-enrichment fraction (below), elution fraction of NIP2 (middle) and elution fraction of MIP2 (above). Three replicas were presented at the same time.

Page – S-23: Figure S15: Number of identified both nonphospho- (blue) and phospho- peptides (red) (a), number of identified His (green), non-localized (purple), Ser (blue), and Thr (orange) phosphopeptides (b) in each fraction. (R=pre-enrichment, L+W=loading+washing and E=pooled elution)

Page – S-23: Figure S16: Boxplot of phospho Histidine peptides in each polymer fraction: Reference (green), MIP2 Load+Wash (yellow), NIP2 Load+Wash (purple), MIP2 Elution (red) and NIP2 Elution (blue).

Page – S-24: Figure S17: The extracted chromatogram of full and identified tryptic pHis peptides (indicated in colors) for 1:1:1 spiking level of BSA:β-casein:Myoglobin digest of (a) elution fraction of MIP3 (b) elution fraction of NIP3 and (c) pre-enrichment fraction, reference.

Page – S-25: Figure S18: LC-MS/MS spectra of four identified phosphorylated histidine peptides. The figures are adapted from Proteome Discoverer version 2.4.

Page – S-26: Figure S19: Number and type of phospho-peptide spectrum matches (PSMs) (a) and corresponding phosphopeptide proportions (b) indicating identified His (green), Ser (blue), Thr (orange) and non-localized (purple) modifications in the elution fractions after extractions using MIP3 and NIP3 or in the pre-enrichment fractions (R) as indicated.

Experimental Section

Materials

Pentaerythritol triacrylate (PETA) was from Polysciences (Warrington, PA, USA). N,N'-azo-bis(2,4-dimethyl)valeronitrile (ABDV) was from Wako Chemicals GmbH (Neuss, Germany); 1-Hydroxybenzotriazole (HOBt), 1,2,2,6,6-pentamethylpiperidine (PMP), Tetrabutylammonium hydroxide solution (TBA.OH) and formic acid (FA) were from Fluka (Deisenhofen, Germany). Trifluoroacetic acid (TFA), acetonitrile (ACN), methanol (MeOH), tetrahydrofuran (THF), ethyl acetate (EtOAc), hexane, acetone, DMSO-d6, $CDCl_3$ and D_2O were from VWR chemicals. Dry dichloromethane (dry DCM), *tert*-butanol (*t*-BuOH) triethylamine (TEA), 2,5-Dihydroxybenzoic acid (DHB), piperidine, acrylamide, bromotrimethylsilane (TMSBr), ammonium bicarbonate (NH_4HCO_3), magnesium sulfate ($MgSO_4$), NaOD (40% in D_2O), hydrogen chloride (HCl) 37%, copper (II) sulfate pentahydrate ($CuSO_4 \cdot 5H_2O$), iodoacetamide (IAA) and 1,4-dithiothreitol (DTT) were from Sigma Aldrich (Milwaukee, USA). Dimethylformamide (DMF), toluene, dry acetonitrile (dry MeCN), dry tetrahydrofuran (dry THF), tiphenylphosphine (PPh_3) and ammonium sufate ($(NH_4)_2SO_4$) were from Acros Organics. N-Ethyldiisopropylamine (DIPEA), (Benzotriazol-1-yloxy)tripyrrolidinophosphonium hexafluorophosphate (PyBOP), sodium azide (NaN_3), sulfuric acid (H_2SO_4), diethyl ether (Et_2O) and dichloromethane (DCM) were from Merck KGaA (Dramstadt, Germany). Diethyl azodicarboxylate (DEAD) was from AlfaAesar (Karlsruhe, Germany). Sodium ascorbate was from AppliChem (Darmstadt, Germany). Empore C8 extraction disk was from 3 M Bioanalytical Technologies (St. Paul, MN, USA). Trypsin was from Promega (Madison, WI, USA).

VHI, VSI, VYI, VpSI, VpYI were custom synthesized by LifeTein, LLC (Hillsborough, NJ, USA). DRVYIHPF was from Fluka (Deisenhofen, Germany). Fmoc-pSerOH, Fmoc-pTyrOH, Fmoc-OSu and L-serine ethyl ester hydrochloride were purchased from Bachem GmbH (Bubendorf, Switzerland). N-3,5-bis(trifluoromethyl)-phenyl-N'-4-vinylphenylurea was synthesized as reported elsewhere.[1] Fmoc-pTyr-OEt and Fmoc-pSer-OEt were synthesized following the protocol reported before.[2] Potassium phosphoramidate was prepared according to procedure reported before by Wei and Matthews.[3] Hydrazoic acid (HN_3) was prepared following the protocol reported before.[4] Diethyl ethynylphosphonate was synthesized according to procedure reported before.[5]

Amino-functionalized macroporous silica beads (NH_2@Si) with an average particle size of 30 μm, a surface area (S) of 45 m^2/g, average pore diameter (D_p) of 47.5 nm, and a pore volume (V_p) of 0.81 mL/g were purchased from Fuji Silysia Chemical Ltd. (Kozoji-cho, Kasugai Aichi, Japan). AcNH@Si was synthesized from NH_2@Si as previously reported.[6]

Apparatus and methods

NMR spectra were recorded using Agilent (Varian) Mercury 400 MHz instrument. Purification of synthesized compounds was performed on preparative HPLC Waters 600 instrument (Waters, Milford MA, USA) equipped with an in-line degasser, a Waters 2487 dual absorbance detector, a Waters 2700 sample manager and a Waters Fraction collector II. A Waters Alliance 2695 analytical HPLC equipped with Photodiode Array Detector 2996 was used in all analytical tests. Mass spectra were recorded on Waters ZQ 2000 LCMS System in positive ion mode. MALDI MS/MS spectra was obtained using a MALDI reflector time of flight mass spectrometer (ultrafleXtreme MALDI TOF/TOF MS/MS; Bruker Daltonics, Bremen, Germany) controlled by flexControl software (version 2.4, Bruker Daltonics, Bremen, Germany). The system was operated in positive ion reflector mode recording MS/MS spectra for m/z 1126.51. Dihydroxybenzoic acid (DHB, 40 mg/mL) in 50% ACN and 1% phosphoric acid was used as matrix. Sample was prepared by mixing 0.5 µL of peptide (10 pmol/µL) with 0.5 µL of matrix. Mass spectrometric data analysis was performed using FlexAnalysis 3.4 software (Bruker Daltonik GmbH, Bremen, Germany).

Experimental Section

Synthesis of Fmoc-pTza-OEt

2-(9H-Fluoren-9-ylmethoxycarbonylamino)-3-hydroxy-propionic acid ethyl ester (Fmoc-Ser-OEt) (**3**) L-serine ethyl ester hydrochloride (1.5 g; 8.90 mmol) and $NaHCO_3$ (1.52 g; 33 mmol) were dissolved in water (100 mL). Next the solution of Fmoc-OSu (2.95 g; 8.90 mmol) in acetone (100 mL) was added, and the resulting reaction mixture stirred at room temperature overnight. The solvents were evaporated and the residue dissolved in ethylacetate (100 mL) and washed with 0.1 M HCl (2 x 50 mL) and water (2 x 50 mL). The organic phase was dried over magnesium sulfate, filtered and the solvent was removed to give product as a white solid (2.85 g, 90%). ^{1}H NMR (400 MHz, DMSO-d6) δ 1.54 (t, 3H), 3.64 (t, 2H); ^{13}C NMR (100 MHz, DMSO-d6) δ 171.18, 156.49, 144.26, 141.17, 128.08, 127.51, 125.71, 125.67, 120.55, 66.18, 61.66, 60.94, 57.18, 47.05, 31.40, 22.51, 14.52, 14.40; MS (ESI+): 356.0638 $[M+H]^+$.

3-Azido-2-(9H-fluoren-9-ylmethoxycarbonylamino)-propionic acid ethyl ester (**4**) A stirred suspension of **3** (2.5 g, 7.0 mmol) and PPh_3 (2.21 g; 8.4 mmol) in dry THF (50 mL) at -78 °C under N_2 was treated with 2.5 M HN_3 in toluene (4.5 mL; 11.3 mmol) followed by dropwise addition of DEAD (1.5 mL; 9.5 mmol). The resulting solution was allowed to warm to room temperature. After 2 h the solution was diluted with water. The layers were separated and the aqueous phase was extracted with Et_2O. The organic phase was dried over magnesium sulfate and filtered. The solvent was evaporated and the residue purified by column chromatography (EtOAc/hexane 3:7) to afford **6** as a white solid (1.45 g, 54%). ^{1}H NMR (400 MHz, $CDCl_3$) δ 1.32 (t, 3H), 3.77 (d, 2H), 4.21-4.32 (m, 3H), 4.37-4.45 (m, 2H), 4.50-4.57 (m, 1H), 5.69 (d, 1H), 7.33 (t, 2H), 7.42 (t, 2H), 7.61 (d, 2H), 7.78 (d, 2H); MS (ESI+): 380.9155 $[M+H]^+$.

3-[4-(Diethoxy-phosphoryl)-[1,2,3]triazol-1-yl]-2-(9H-fluoren-9 ylmethoxycarbonylamino)-propionic acid ethyl ester (**5**) $CuSO_4$ x 5 H_2O (10 mol %) was added to a solution of the azide **4** (0.25 g; 0.67 mmol), diethyl ethynylphosphonate (0.11 g; 0.67 mmol) and sodium ascorbate (10 mol %) in 1:1 *t*-BuOH/water (10 mL). The resulting solution was stirred overnight. After the reaction was finished the solution was diluted with water (20 mL), and extracted with DCM. The organic phase was washed with brine (20 mL), dried and evaporated, and the residue was subjected to flash chromatography (EtOAc/hexane 9:1) to afford the product as a white solid (0.21 g, 59%). ^{1}H NMR (400 MHz, $CDCl_3$) δ; ^{31}P NMR (162 MHz, $CDCl_3$) δ 6.22. MS (ESI+): 564.877 $[M+Na]^+$.

3-[4-Phosphono-[1,2,3]triazol-1-yl]-2-(9H-fluoren-9-ylmethoxycarbonylamino)-propionic acid ethyl ester (**2**) To a solution of **5** (0.15 g; 0.28 mmol) in DCM (5 mL), TMSBr (0.36ml; 2.80 mmol) was added and the resulting solution was stirred at room temperature for 24h. Next the solvent was removed by a rotary evaporator. Residue was dissolved in 10 mL MeOH and the mixture was stirred for another 2 h. The product precipitated out form the solution. Solvent was evaporated and residue was dried in vacuo to afford product as a white solid (0.13 g, 97%) . ^{1}H NMR (400 MHz, DMSO-d6) δ 1.14 (t, 3H) 4.09 (q, 2H), 4.20-4.33 (m, 3H), 4.56-4.62 (m, 1H), 4.67-4.85 (m, 2H), 7.34 (t, 2H), 7.41 (t, 2H), 7.67 (t, 2H), 7.89 (d, 2H), 8.04 (d, 1H), 8.22 (s, 1H); ^{13}C NMR (100 MHz, DMSO-d6) δ 169.72, 156.30, 144.15, 144.04, 142.99, 141.14, 140.71, 130.62, 130.30, 128.10, 127.56, 125.62, 120.56, 66.35, 61.67, 54.50, 49.47, 46.94, 14.33; ^{31}P NMR (162 MHz, DMSO-d6) δ 2.32. MS (ESI+): 486.8279 $[M+H]^+$.

Synthesis of pHis reference peptides

Briefly, VHI (10 mg) or DRVYIHPF (5 mg) was dissolved in 10 mM NH_4HCO_3 pH=8 (1 mL) followed by addition of potassium phosphoramidate (100 mg). This was stirred at room temperature overnight. VpHI or DRVYIpHPF, respectively was isolated from the mixture by preparative HPLC using XBridge™ Prep C18 5 µm OBD 19 x 100 mm column (Waters, Milford, MA, USA). The mobile phases were (A) 10mM NH_4HCO_3 and (B) ACN/10mM NH_4HCO_3 (9:1). The chromatography was performed using a linear gradient from 0% B to 35% B in 60 min and a flow rate of 5 mL/min. The detection was performed by UV absorbance measurement at 210 nm. The identity of peptides was confirmed by electrospray mass spectrometry (VpHI ESI+ 447.8923; DRVYIpHPF ESI+ 1125.9938). The NMR spectra of VHI and VpHI peptides was recorded in D_2O with the pH adjusted to a value of 10 with NaOD (Figure S). MALDI MS/MS spectra of DRVYIpHPF was recorded for precursor ion *m/z*=1126.505 using DHB matrix (Figure S).

The VEI peptide was synthesized by standard Fmoc solution phase peptide synthesis. Each coupling step was performed using PyBOP/DIEA/HOBt reagents in the molar ratio of 1.1/2.2/1 in DMF. Fmoc deprotection was performed using 20% piperidine in DMF. Removal of the *tert*-butyl ester group was carried out using 95% TFA and deprotection of the methyl ester using LiOH. The intermediate products and the final product were purified by preparative HPLC on J'sphere ODS-H80 100 x 20 mm column (YMC Europe GmbH, Dinslaken, Germany). The mobile phases were (A) H_2O + 0.1% TFA and (B) ACN + 0.1% TFA. The chromatography was performed using a linear gradient from 5% B to 95% B in 30 min and a flow rate of 5 mL/min. The detection was performed by UV absorbance measurement at 210 nm.

References

[1] Hall, A. J.; Manesiotis, P.; Emgenbroich, M.; Quaglia, M.; Lorenzi De, E.; Sellergren, B. *J. Org. Chem.* **2005,** *70(5)*, 1732-1736.

[2] Wierzbicka, C.; Liu, M.; Bauer, D.; Irgum, K.; Sellergren, B. *J. Mater. Chem. B* **2017,** *5*, 953-960.

[3] Wei, Y. F.; Matthews, H. R. *Methods Enzymol.* **1991,** *200*, 388-414.

[4] Yeager, A. R.; Finney, N. S. *J. Org. Chem.* **2004,** *69(3)*, 613-618.

[5] Mukai, S.; Flematti, G. R.; Byrne, L. T.; Besant, P. G.; Attwood, P. V.; Piggot, M. J. *Amino Acids* **2012,** *43*, 857-874.

[6] Wierzbicka, C.; Torsetnes, S. B.; Jensen, O. N.; Shinde, S.; Sellergren, B. RSC Adv. **2017,** *7(28)*, 17154-17163.

Tables

Table S1: Prepolymerization solution compositions and formats used to prepare imprinted and non-imprinted polymers.

**Polymer**	Format	Template	Host monomer	Crosslinker
MIP1	Crushed monolith	Fmoc-pTza-OEt • PMP (0.5)	FM (1)	PETA (13.3)
MIP2	Crushed monolith	Fmoc-pTza-OEt • TBA-OH (0.5)	FM (1)	PETA (13.3)
MIP3	Microspheres	Fmoc-pTza-OEt • TBA-OH (0.5)	FM (1)	PETA (13.3)
NIP1	Crushed monolith	-	FM (1)	PETA (13.3)
NIP2	Crushed monolith	-	FM (1)	PETA (13.3)
NIP3	Microspheres	-	FM (1)	PETA (13.3)

Table S2: Imprinting Factors (IF) for Fmoc-protected templates corresponding to the data in Fig. 2.

Binding Condition (+0.1% TEA)	**Fmoc-pTza-OEt**	Fmoc-pSer-OEt	Fmoc-pTyr-OEt
95% MeCN	**37.9**	25.8	4.1
90% MeCN	1.1	3.0	6.5
75% MeCN	1.0	1.1	3.3
50% MeCN	1.0	1.0	1.6
25% MeCN	1.1	1.2	1.0
5% MeCN	1.0	1.0	1.1

Table S3: Imprinting Factor (IF) for tri-peptides corresponding to the data in Fig. 3.

Binding Condition (+0.1% TEA)	VpSI	**VpHI**	VEI	VHI
95% MeCN	4.3	**4.7**	1.8	1.5
90% MeCN	2.8	3.8	1.4	1.4
75% MeCN	1.7	2.9	1.3	2.0
50% MeCN	1.3	1.1	1.0	1.1
25% MeCN	1.4	1.6	1.4	1.5
5% MeCN	2.2	1.5	1.4	1.2

Table S4: List of identified phosphohistidine peptides

Sequence	Missed Cleavages	Modifications	m/z	Charge	Number of PSMs	Delta m/z (ppm)	Mascot Score	Mascot Delta Score	Confidence MIP	Confidence NIP	Confidence Reference
**VEADIAGHGQEVLIR**	0	1xPhospho [H8(100)]	843.91	2	28	-0.93	107	1	High	High	High
**HPGDFGADAQGAMTK**	0	1xPhospho [H1(100)]	791.82	2	20	-1.26	86	1	High	High	High
**GHHEAELKPLAQSHATK**	0	1xPhospho [H3(99.6)]	645.31	3	70	-1.48	80	0.05	High	High	Peak Found
**HPGDFGADAQGAMTK**	0	1xPhospho [H1(100)]; 1xOxidation [M13]	799.81	2	30	-1.12	72	1	High	High	High
**KGHHEAELKPLAQSHATK**	1	2xPhospho [H3(100); H4(100)]	714.66	3	12	-1.6	65	0.0923	High	Peak Found	Peak Found
**GHHEAELKPLAQSHATK**	0	2xPhospho [H3(100); H14(100)]	671.96	3	25	-0.76	58	0.0172	High	Peak Found	Peak Found
**LFTGHPETLEK**	0	1xPhospho [H5(100)]	676.31	2	44	-1.93	52	0.3077	High	High	High
**HPGDFGADAQGAMTK**	0	1xDeamidated [Q10]; 1xPhospho [H1(100)]	792.31	2	2	3.29	30	0.7333	High	Not Found	Peak Found
**GHHEAELKPLAQSHATK**	0	3xPhospho [H2(100); H3(100); H14(100)]	698.62	3	3	-2.11	30	0.1	High	Peak Found	Peak Found
**KGHHEAELKPLAQSHATK**	1	3xPhospho [H3(100); H4(100); S/H/T]	741.32	3	4	-1.27	24	0.0417	High	Not Found	Peak Found

Figures

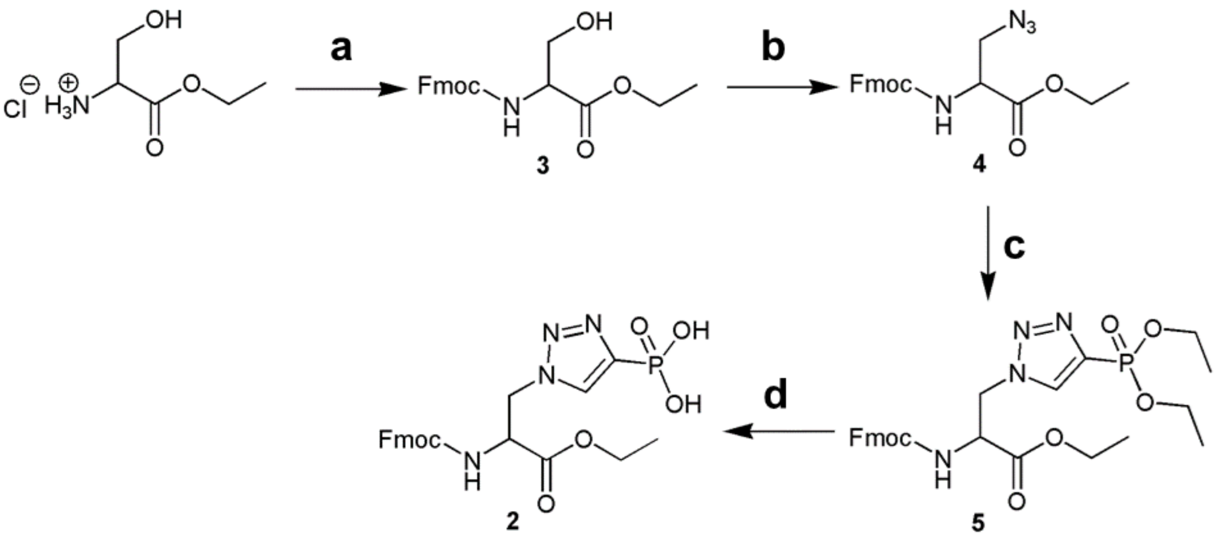

Figure S1. Synthesis of pHis analogue template Fmoc-pTza-OEt (**2**). a) Fmoc-OSu, $NaHCO_3$, acetone/water; b) DEAD, PPh_3, HN_3, THF; c) diethyl ethynylphosphonate, $CuSO_4$ x 5 H_2O, sodium ascorbate, tBuOH/water; d) 1. TMSBr, DCM, 2. MeOH.

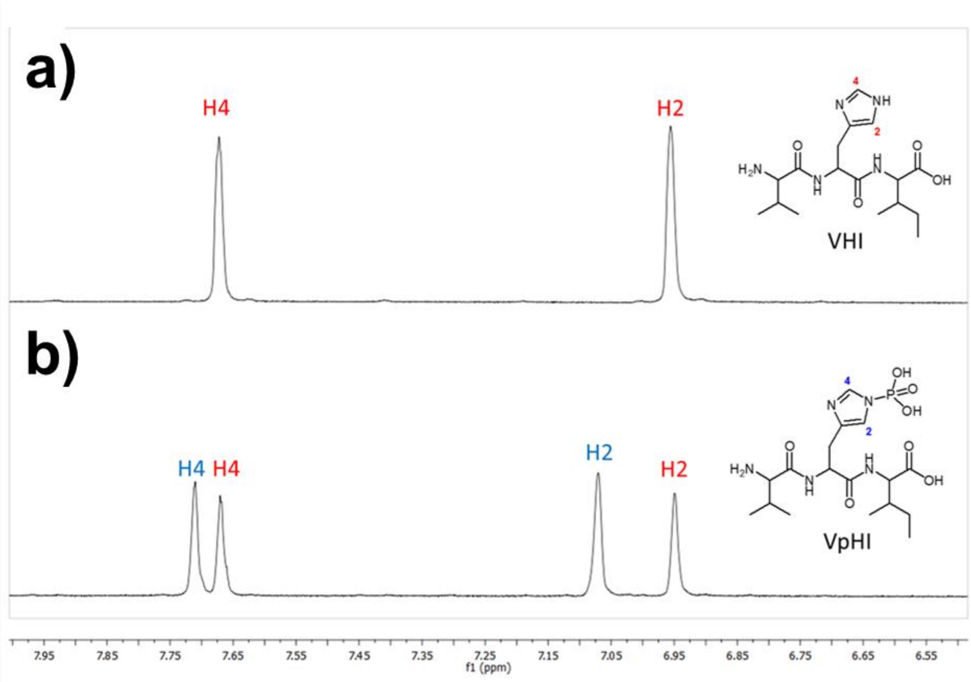

Figure S2. Portion of NMR spectra (6.5-8.0 ppm) of (a) VHI and (b) VpHI (partially dephosphorylated) in D_2O at pH 10.

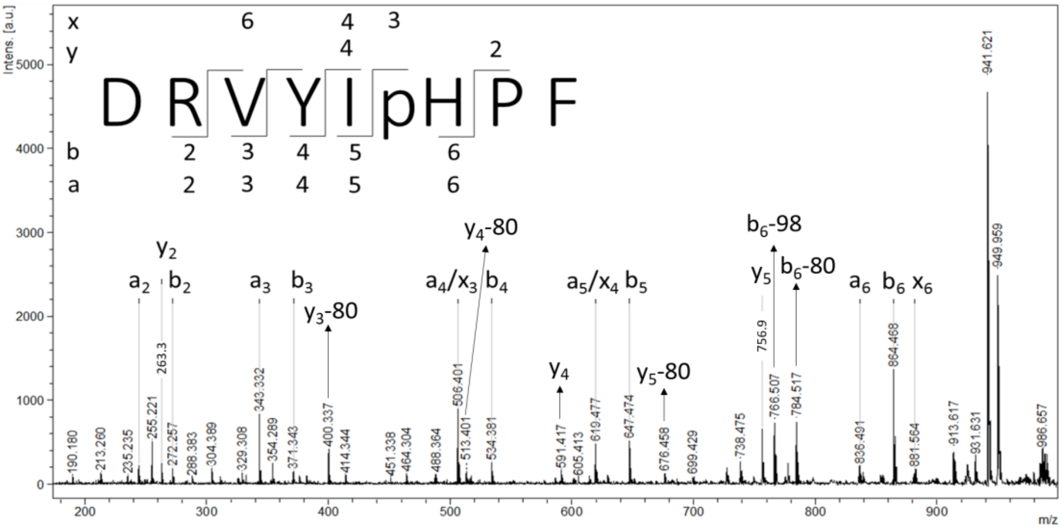

Figure S3. MALDI MS/MS spectra of DRVYIpHPF peptide (precursor ion *m/z* = 1126.505)

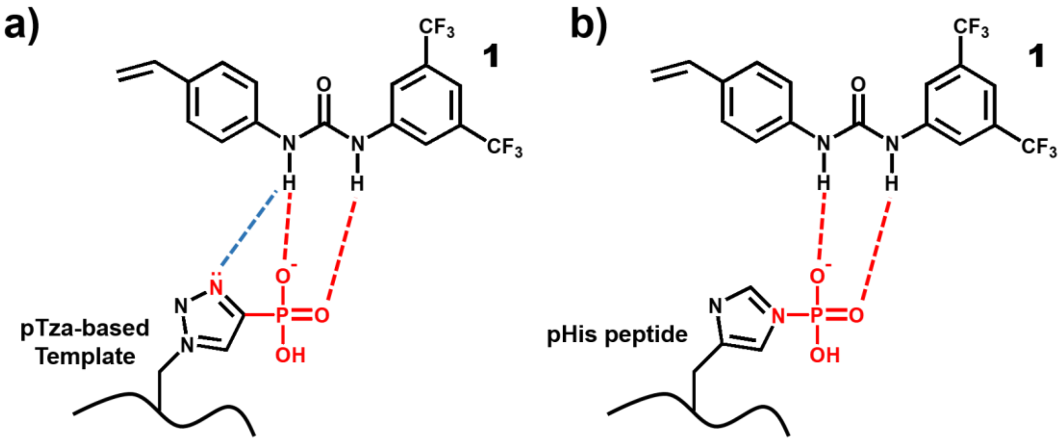

Figure S4. Hydrogen bonding association of urea monomer, **1** with pTza-based template (a) and pHis peptide (b).

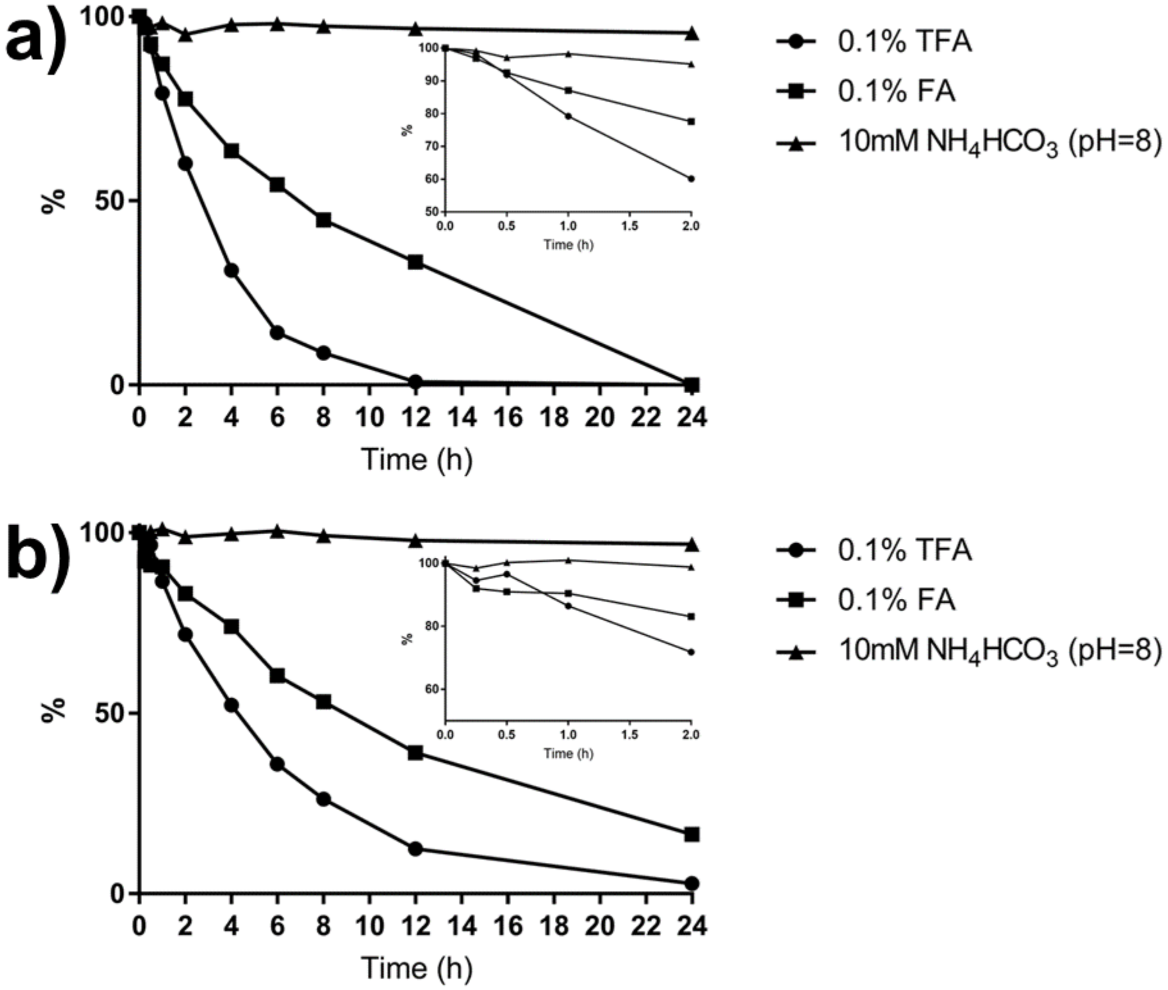

Figure S5. Stability profile of (a) VpHI and (b) DRVYIpHPF in 0.1% TFA, 0.1% FA and 10mM NH_4HCO_3 (pH=8).

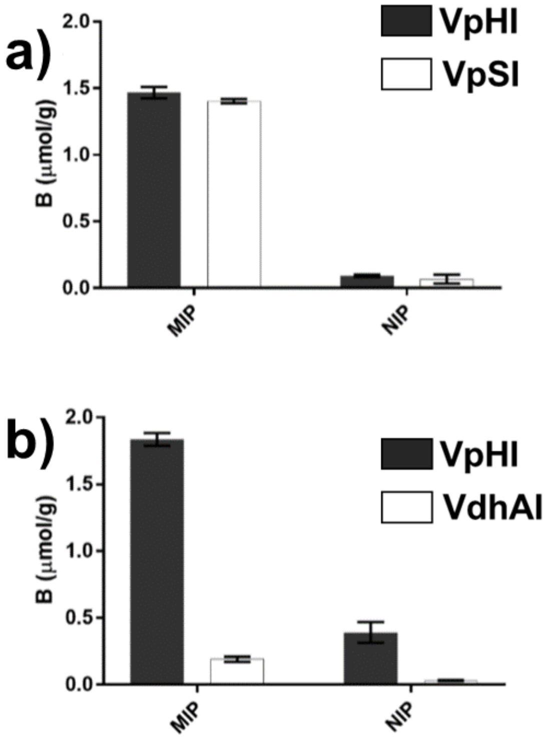

Figure S6. HPLC-UV chromatograms from a binding equilibrium test of an equimolar mixture of (a) VpHI and VpSI, (b) VpHI and VdhAI (resulting from dephosphorylation of VpSI) in 95% ACN + 0.1% TEA in presence of MIP1 and NIP1. The bars show the average of three replicas and the error bars represent the corresponding standard deviation.

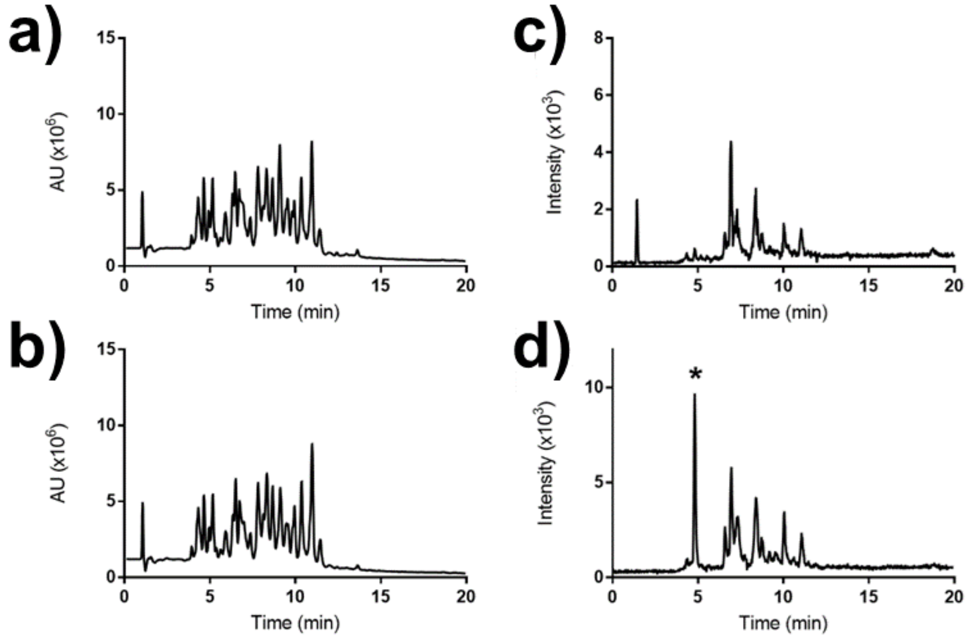

Figure S7. HPLC-UV chromatograms (a, b) and corresponding ESI-MS selective ion monitoring (SIM, m/z=447.5) spectra (c, d) of the flow through + washing fractions from MIP1 (a, c) and NIP1 (b, d) after loading a sample of digested BSA (1 µmol) spiked with VpHI (0.2 µmol). The signal from VpHI is marked with an asterisk.

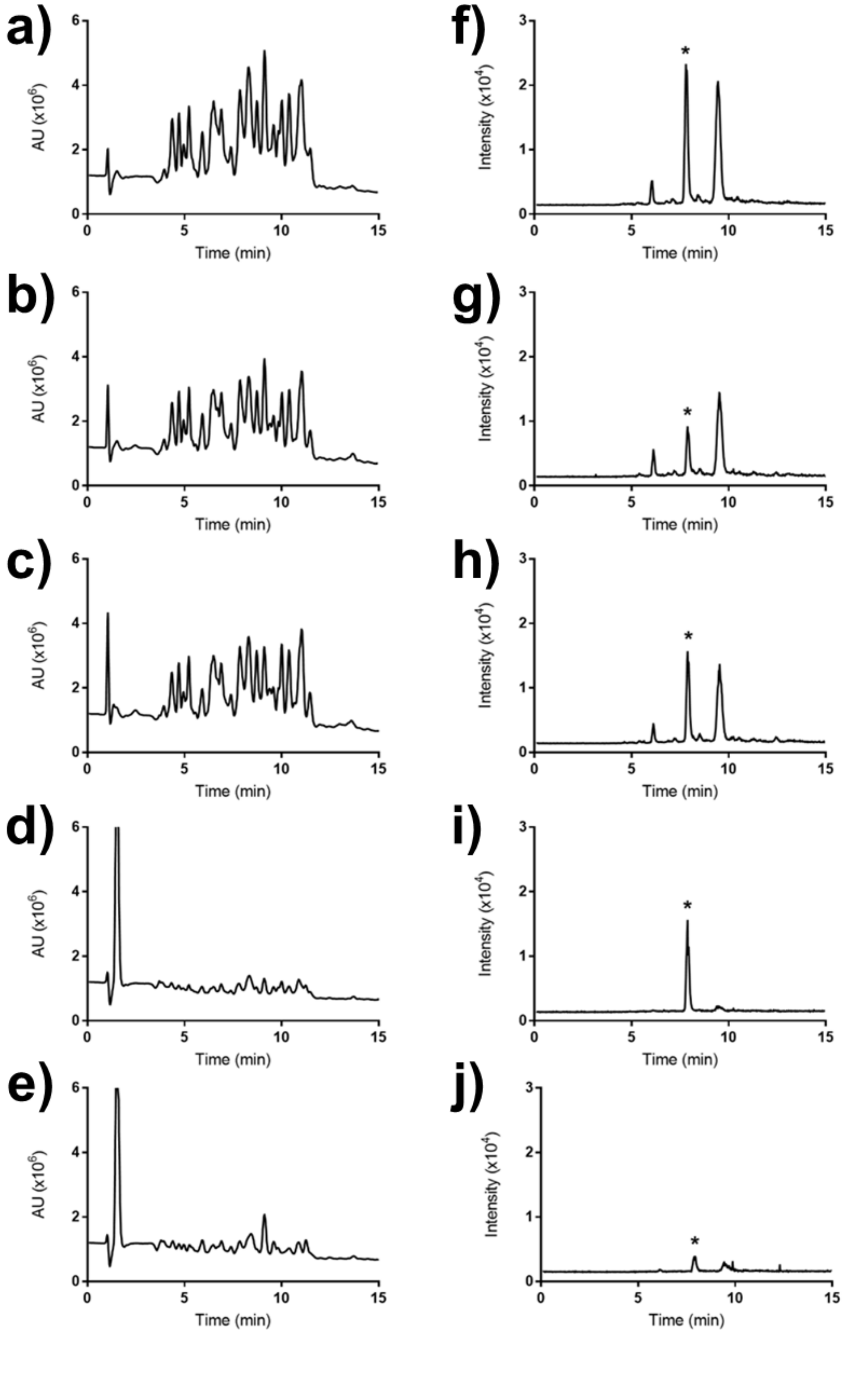

Figure S8. HPLC-UV chromatograms (a-e) and corresponding ESI-MS selective ion monitoring (SIM, m/z=1126.5) spectra (f-j) of a sample of digested BSA (1 µmol) spiked.with DRVYIpHPF (0.2 µmol) before enrichment (a, f), and the flow through + washing fractions from MIP1 (b, g) and NIP1 (c, h) and elution fractions from MIP1 (d, i) and NIP1 (e, j) after solid phase extraction. The signal from DRVYIpHPF is marked with an asterisk.

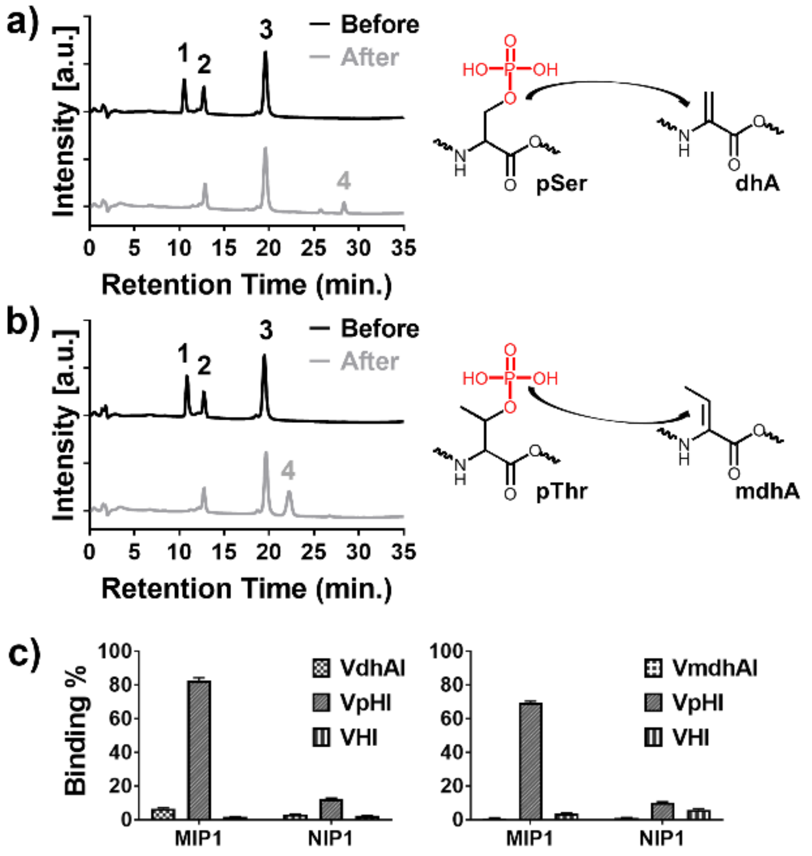

Figure S9: HPLC-UV chromatogram of p-peptide mixtures containing VpSI (a) and VpTI (b) before and after β–elimination (0.1 M $Ba(OH)_2$ @50 °C for 24 h). VpSI and VpTI=1, VpHI=2, VHI=3, VdhAI and VmdhAI=4. Binding degree of VdhAI (left) and VmdhAI (right) in the presence of VpHI and VHI on MIP1 and NIP1 in 95% MeCN + 0.1% TEA (c).

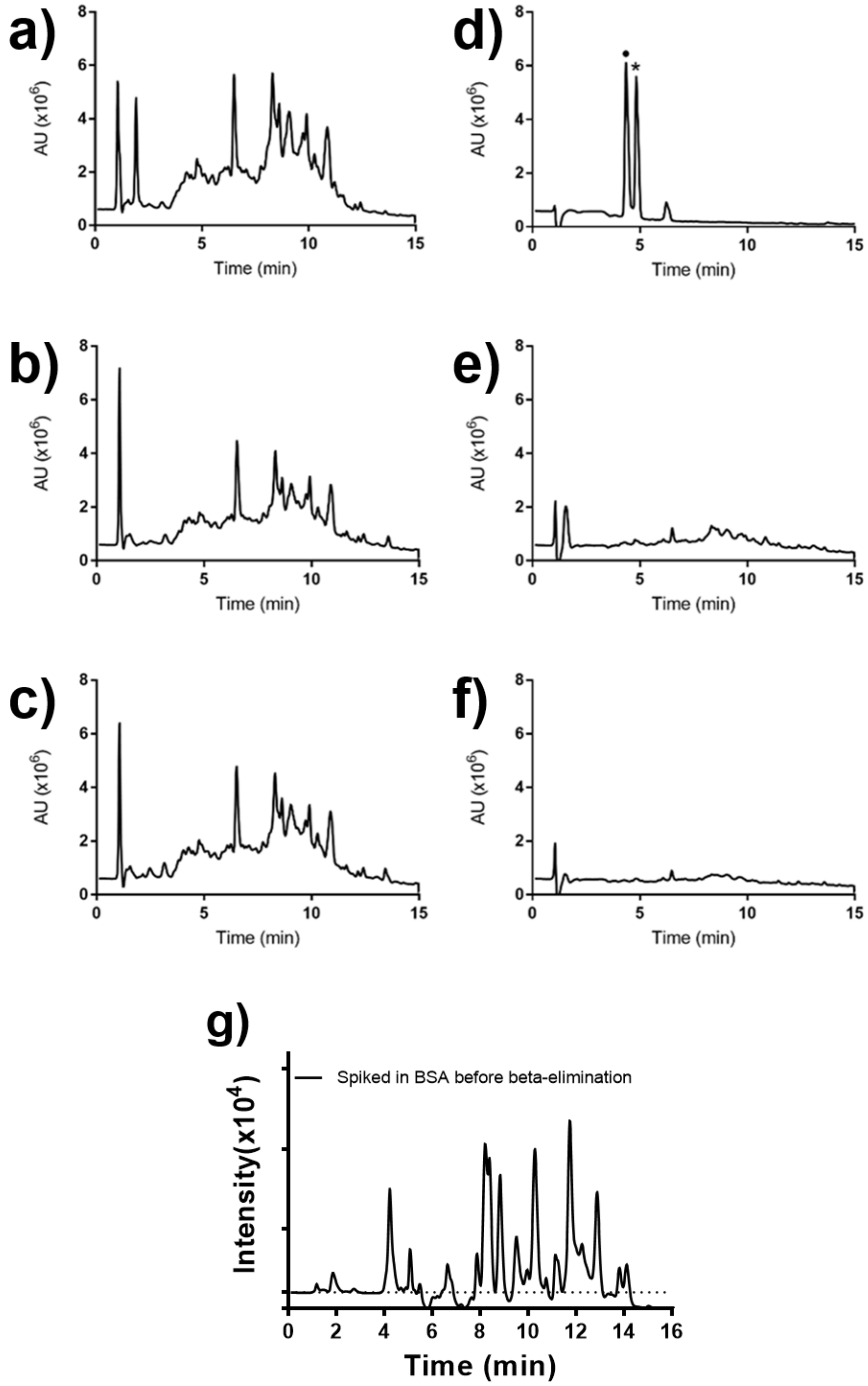

Figure S10. HPLC-UV chromatograms of a sample of digested BSA (1 µmol) spiked with VpSI and VpHI (0.2 µmol each) and treated with $Ba(OH)_2$ (a), the combined flow through and washing fractions from MIP1 (b) and NIP1 (c), a mixture of VpSI and VpHI (d) and the elution fraction from MIP1 (e) and NIP1 (f) and the BSA digest with spiked peptides before beta-elimination (g). The signals from VpSI and VpHI are marked with a dot and asterisk, respectively.

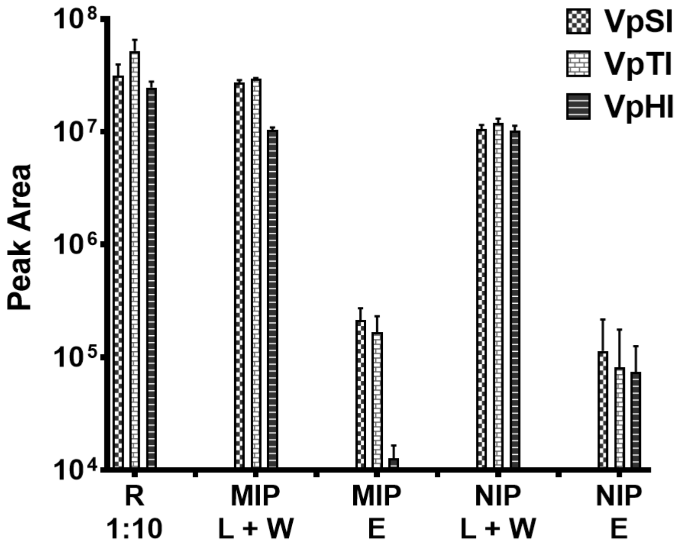

Figure S11: Peak areas of the three phosphorylated tripeptides obtained from each SPE fraction in MIP1 and NIP1 fractions of 1:10 spiking level. (R=pre-enrichment, L+W=loading+washing, E=pooled elution)

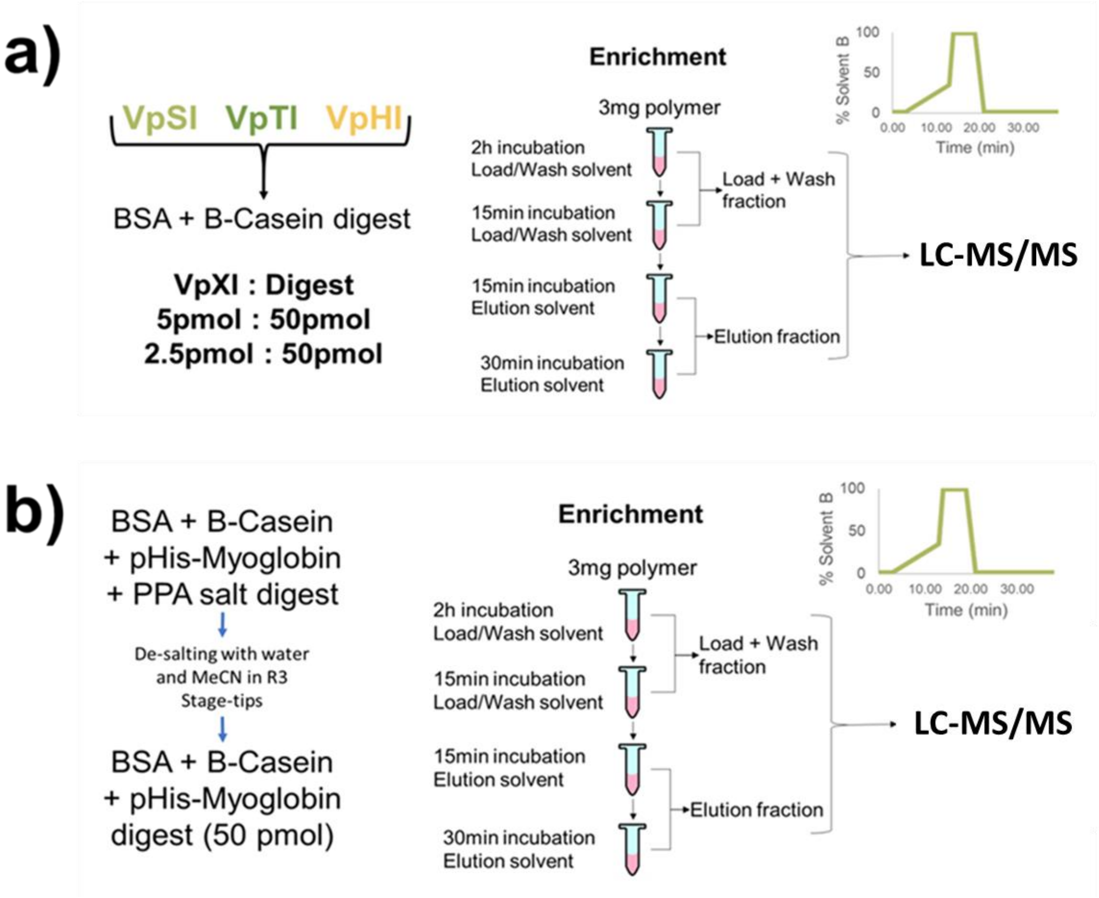

Figure S12: Schematic representation of the workflows. a) Three short phosphopeptides were first spiked in BSA+ B-Casein digest in two spiking levels (1:10 and 1:20) before SPE. Load+Wash and Elution fractions were collected and dried prior LC-MSMS analysis. b) Protein digests were first de-salted and dried prior MIP SPE. Load+Wash and Elution fractions were collected and dried prior LC-MS/MS analysis.

a)

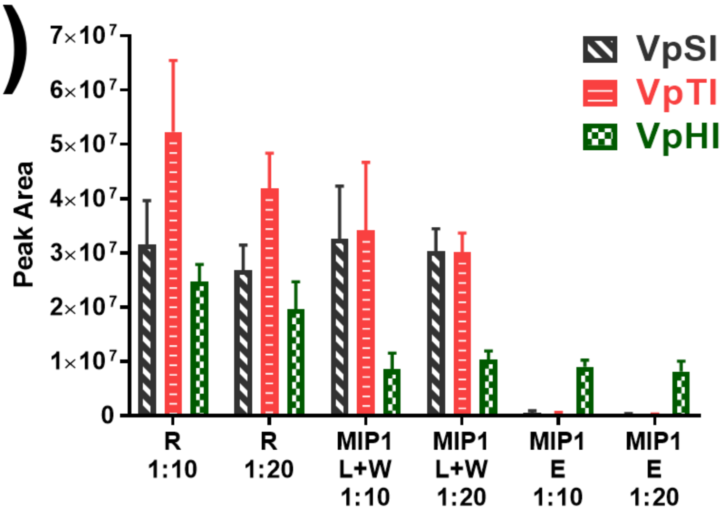

b)

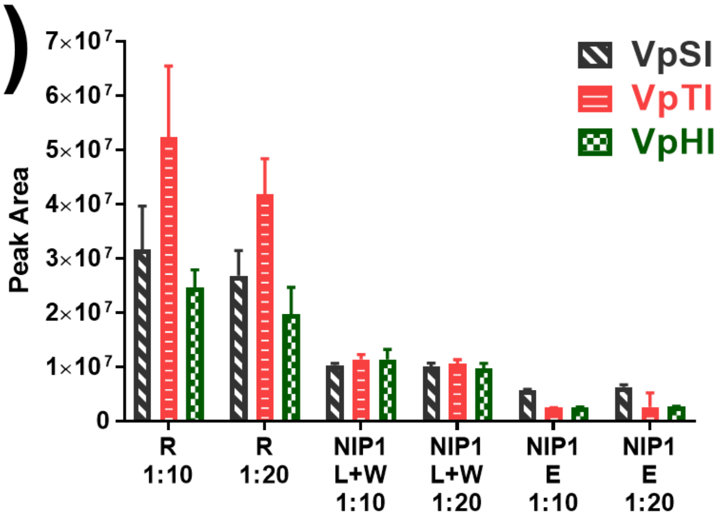

Figure S13: Peak areas of the three phosphorylated tripeptides obtained from each SPE fractions obtained in MIP1 (a) and NIP1 (b) at 1:10 and 1:20 spiking level (R=reference, L+W=loading+washing, E=pooled elution).

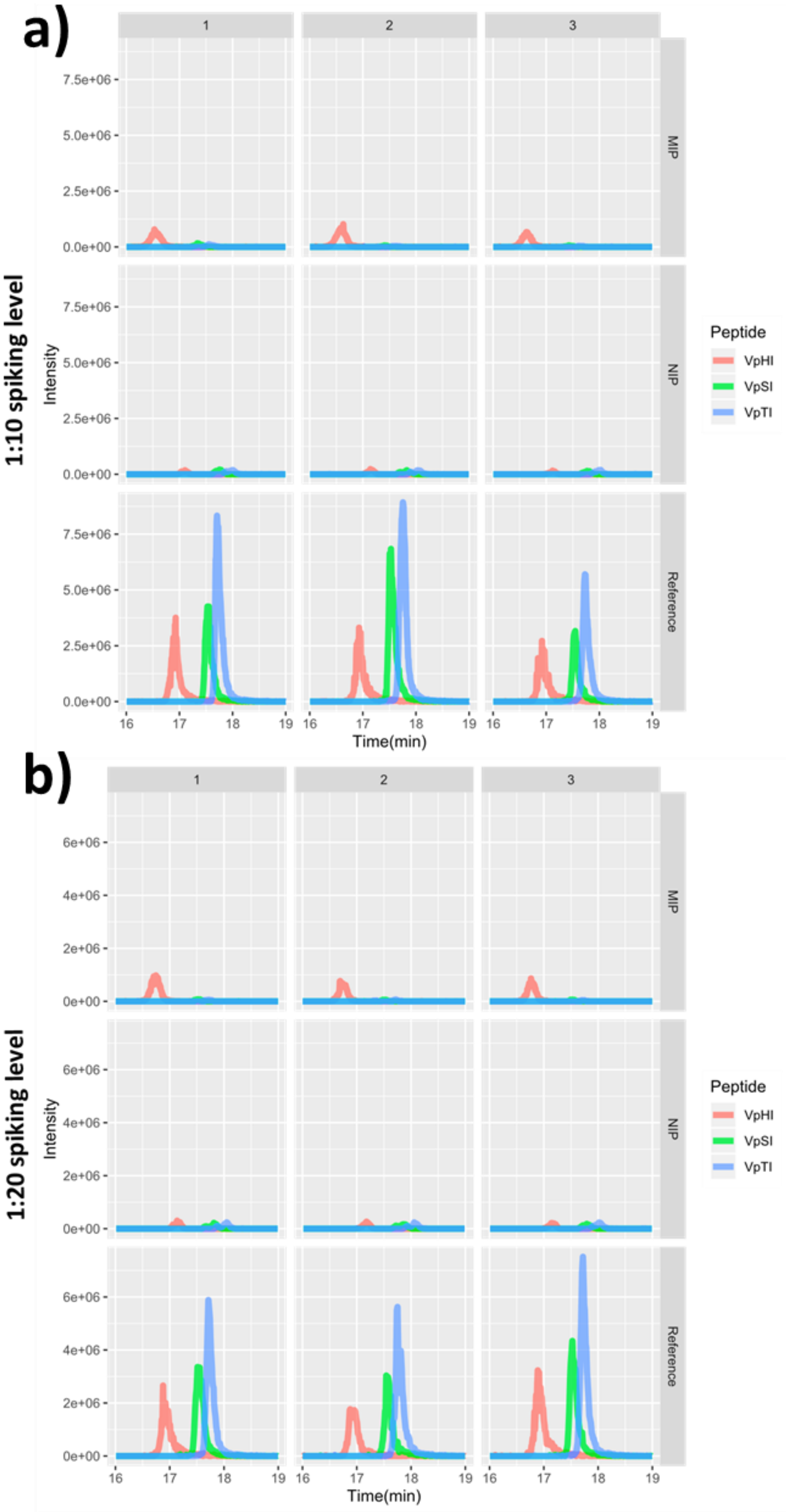

Figure S14: The extracted chromatogram of tripeptides (VpHI, VpTI, and VpSI) for 1:10 spiking level (a) and 1:20 spiking level (b) of pre-enrichment fraction (below), elution fraction of NIP2 (middle) and elution fraction of MIP2 (above). Three replicas were presented at the same time.

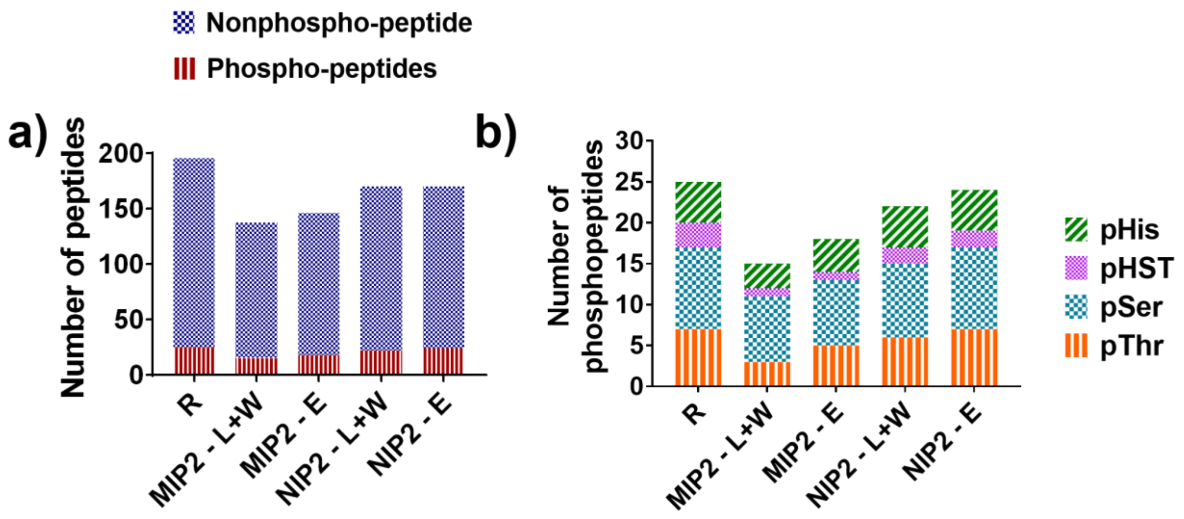

Figure S15: Number of identified both nonphospho- (blue) and phospho- peptides (red) (a), number of identified His (green), non-localized (purple), Ser (blue), and Thr (orange) phosphopeptides (b) in each fraction. (R=pre-enrichment, L+W=loading+washing and E=pooled elution)

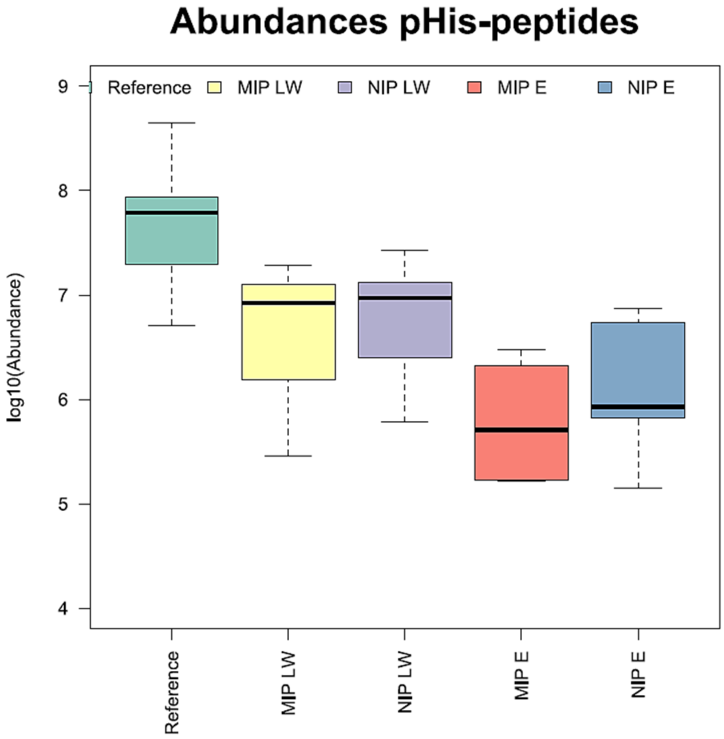

Figure S16: Boxplot of phospho Histidine peptides in each polymer fraction: Reference (green), MIP2 Load+Wash (yellow), NIP2 Load+Wash (purple), MIP2 Elution (red) and NIP2 Elution (blue).

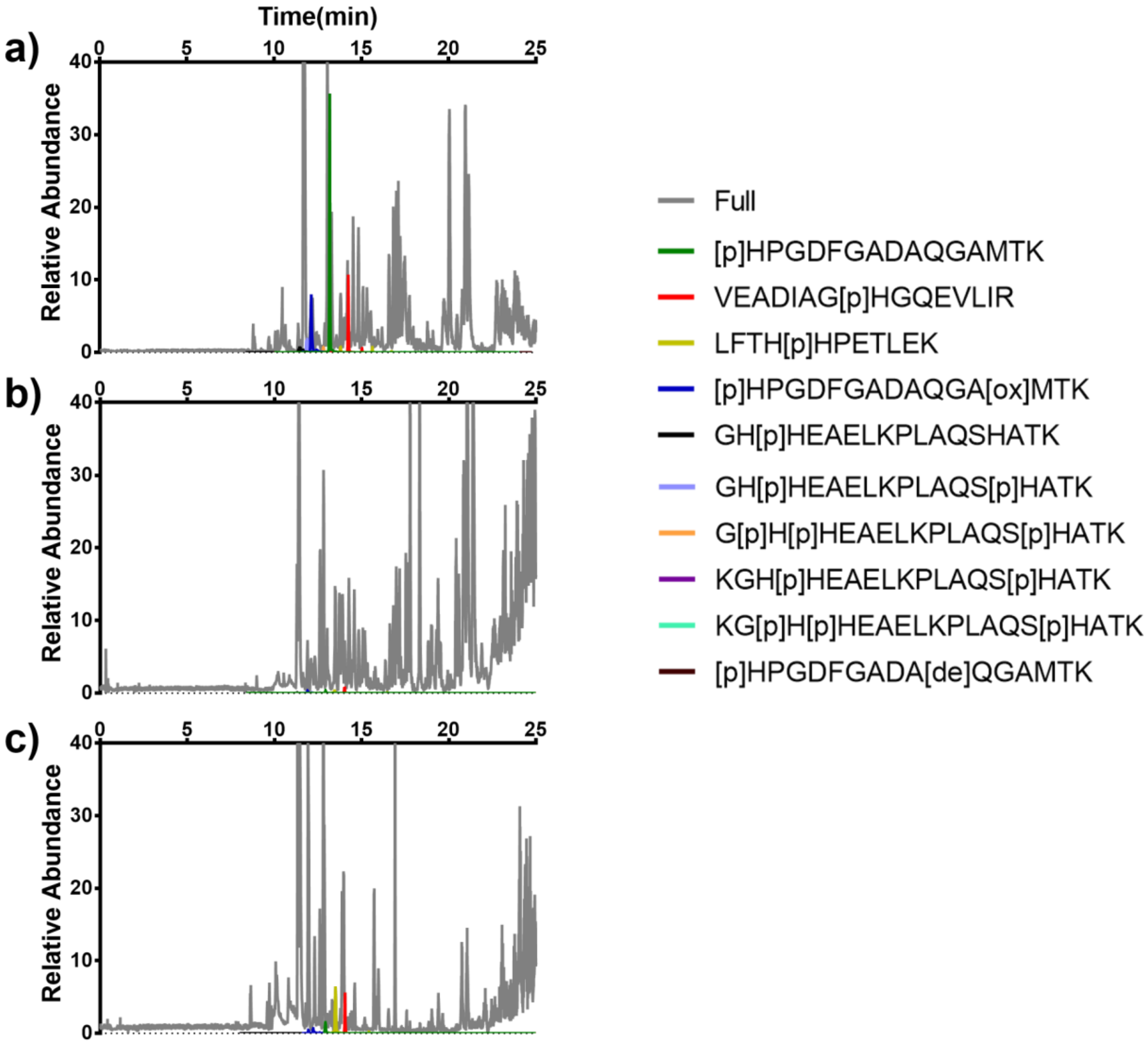

Figure S17: The extracted chromatogram of full and identified tryptic pHis peptides (indicated in colors) for 1:1:1 spiking level of BSA:β-casein:Myoglobin digest of (a) elution fraction of MIP3 (b) elution fraction of NIP3 and (c) pre-enrichment fraction, reference.

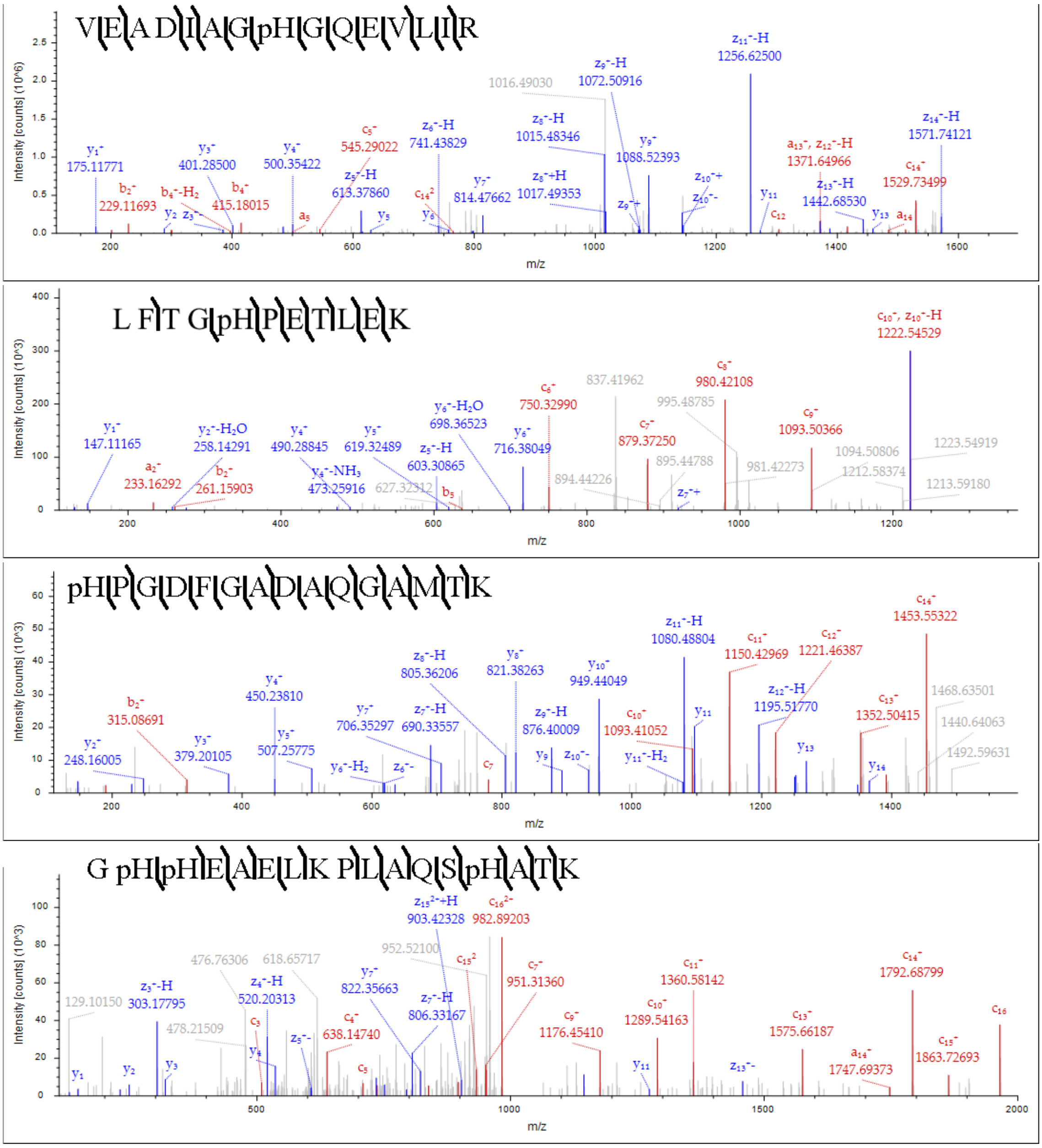

Figure S18: LC-MS/MS spectra of four identified phosphorylated histidine peptides. The figures are adapted from Proteome Discoverer version 2.4.

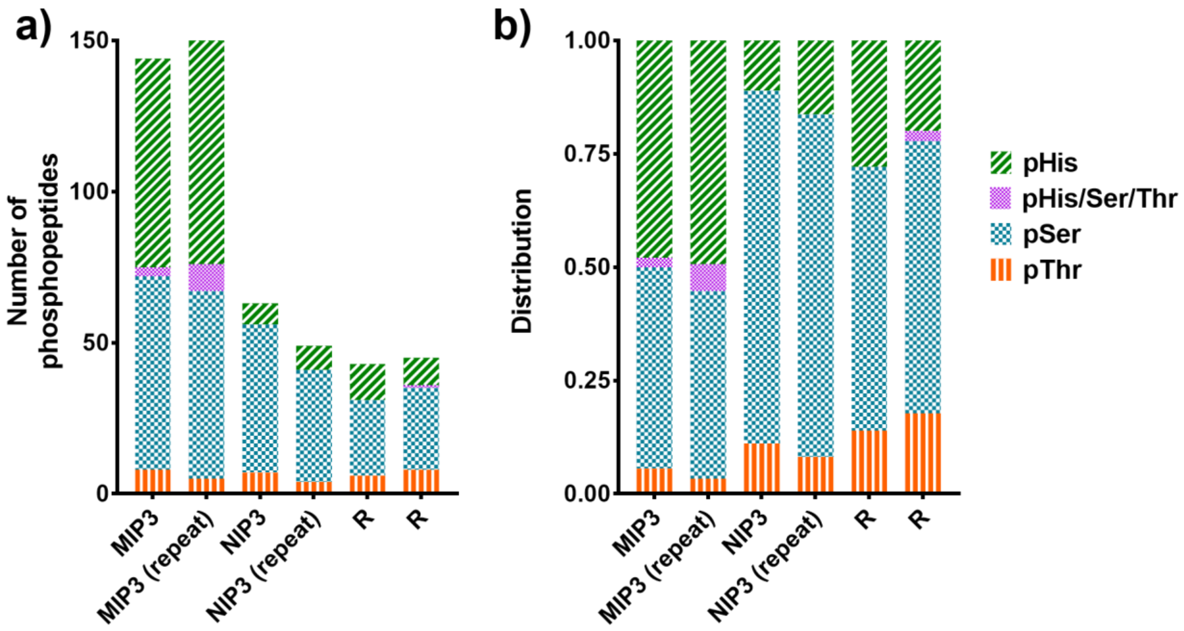

Figure S19: Number and type of phospho-peptide spectrum matches (PSMs) (a) and corresponding phosphopeptide proportions (b) indicating identified His (green), Ser (blue), Thr (orange) and non-localized (purple) modifications in the elution fractions after extractions using MIP3 and NIP3 or in the pre-enrichment fractions (R) as indicated.
